# A novel mechanical-laser collaborative intra-row weeding prototype: structural design and optimization, weeding knife simulation and laser weeding experiment

**DOI:** 10.3389/fpls.2024.1469098

**Published:** 2024-10-30

**Authors:** Rui Hu, Long-Tao Niu, Wen-Hao Su

**Affiliations:** College of Engineering, China Agricultural University, Beijing, China

**Keywords:** mechanical weeding, laser weeding, structure design, EDEM, weeding device, slider-crank mechanism

## Abstract

**Introduction:**

The competition between intra-row weeds and cultivated vegetables for nutrients is a major contributor for crop yield reduction. Compared with manual weeding, intelligent robots can improve the efficiency of weeding operations.

**Methods:**

This study proposed a novel mechanical-laser collaborative intra-row weeding device structure. A slider-crank mechanism size optimization algorithm was proposed, and the correctness of the algorithm was verified by ADMAS software. Finally, the crank and link lengths were determined to be 87 mm and 135 mm, respectively. The resistance of triangular weeding knives with different penetration angles and edge angles in the soil was simulated and analyzed using EDEM software. The simulation results show that the triangular weeding knife with a soil penetration angle of 0 ° and an edge angle of 30 ° encountered the least resistance. In addition, weed control experiments with different powers and lasers were conducted using 200 W NIR and 200 W blue lasers. The experimental results show that the time it took for a 50 W blue laser and a 100 W NIR laser to remove small weeds was approximately between 0.3 and 0.4 s, and the time it took for a 50 W blue laser to remove larger weeds was approximately between 0.5 and 0.6 s. The time it took for 75 W and 50 W NIR lasers to remove weeds was more than 1 s.

**Results:**

Based on the above research results, a prototype of a mechanical-laser collaborative intra-row weeding device was successfully built.

**Discussion:**

This study provides a new idea for the field of intelligent weeding. The simulation and experimental results can provide a reference for the research and development of mechanical weeding and laser weeding equipment.

## 1 Introduction

Field weeds compete with vegetables such as lettuce for resources, resulting in a decrease in vegetable yields. Therefore, the removal of weeds from fields is crucial to increase vegetable yields. Field weed removal is mainly divided into inter-row weeding and intra-row weeding. Inter-row weed removal has become mature because it does not require avoiding vegetable seedlings and is technically easy. Intra-row weed removal is technically difficult because it requires protecting vegetable seedlings from being harmed. It is a research difficulty in the field of weeding. The main methods of intra-row weed removal include manual weeding, chemical weeding, mechanical weeding and laser weeding. Manual weeding is inefficient and costly. Herbicides are widely used to control weeds due to their low cost and effectiveness (Raja et al., 2020a, 2020b; Wu et al., 2020; Alanaz et al., 2023; El-Mastouri et al., 2024; Li et al., 2023; Liu et al., 2024; Pingarron-Cardenas et al., 2024; Xie et al., 2024; Zhang et al., 2023). Long-term use of herbicides can cause serious environmental pollution (Tudi et al., 2021). Environmentally friendly weeding methods such as mechanical weeding and laser weeding have become research hot spots in recent years. In addition, Michaliszyn-Gabry´s research shows that farmers have a positive attitude towards the introduction of new pollution-free weed control technologies such as laser weeding (Michaliszyn-Gabryś et al., 2024a, 2024b).

Mechanical weeding has many advantages, including being environmentally friendly and suitable for fields with any weed density. Dedousis and Godwin (2008) proposed a disc weeding knife for intra-row weed removal, established a mathematical model of the disc weeding knife, and obtained the appropriate size parameters of the disc weeding knife. They also built a prototype for field experiments. The experimental results showed that the weeding device can remove 62-87% of field weeds (Tillett et al., 2008). Sellmann et al. (2014) had developed a robot called BoniRob for intra-row weed removal. The BoniRob robot uses a parallel mechanism with four degrees of freedom (one rotational and three translational) to control the tube-stamp weeding tool (Langsenkamp et al., 2014). Pérez-Ruíz et al. (2014) proposed a hydraulically driven triangular weeding knife and developed a weeding robot for weed removal in tomato intra-row. Experimental results showed that the weeding robot can reduce the manual weeding workload by 57.5%. Saber et al. (2015) developed an automated mechanical intra-row weeding machine prototype based on a pair of rotating pinch-roller weeding mechanisms for intra-row weeding of large spacing vegetable crops. The test results showed that the control effects of the weeding device on *Southern crabgrass*, *Benghal dayflower* and *Purple nutsedge* were 33.9%, 18.3% and 5.4% respectively. Jiang et al. (2023) developed an intra-row weeding system based on a vision system and opening and closing weeding knives. The weeding system successfully removed weeds intra-row at a speed of 3.28 km/h with an accuracy of 80.25%. With the development of technology, the weed control rate of mechanical weeding can reach more than 80%, but it is still impossible to completely remove the intra-row weeds. Ju et al. (2024) proposed a rice field weeding robot based on MW-YOLOv5s. The results of field experiments showed that the weeding rate was 82.4% and the seedling injury rate was 2.8%. Jiao et al. (2024) designed a double-layer elastic rod intra-row weeding device that can press weeds into the soil while avoiding damage to rice. Field tests were conducted under experimental conditions of a weeding depth of 15 mm and a weeding speed of 0.9 m/s. The test results showed that the optimal position of the adjustment mechanism was 270 mm, the weeding rate was 80.65%, and the seedling injury rate was 3.36%. The weeds that are not removed are mainly distributed around crops such as vegetables. Removing this part of weeds can easily damage crops. How to use a weeding knife to remove weeds around crops without damaging vegetables and other crops is a difficult problem that mechanical weeding needs to solve now. Quan et al. (2022) developed a new deep learning-based intelligent intra-row mechanical robot weeding system for crop and weed detection. Three types of weeding knives were designed. Field test results showed that among the three types of weeding knives, plow weeding knives were most suitable for flat plowing and wedge-shaped weeding knives were most suitable for ridge farming. The final weeding rate was 85.91%, and the crop damage rate was 1.17%. The rotary weeding knife further improves the weeding rate of mechanical weeding, but the weeding rate of mechanical weeding still has a lot of room for improvement.

Laser technology is widely used in fields such as medicine, material processing, and radioactive contaminated surface decontamination. Due to its mature basic theory and excellent performance, many scholars have devoted themselves to the application of laser in intra-row weeding in recent years (Andreasen et al., 2022; Zhang et al., 2024). Marx et al. (2012) studied the effects of CO_2_ laser radiation (10,600 nm) on three growth stages of *Amaranthus aurantia* and *Amaranthus retroflexes* under 3 spot diameters, 3 spot positions and 6 laser intensities. Experimental results showed that the fresh weight of weeds dropped by 90% after two weeks of irradiation. Rakhmatulin and Andreasen (2020) designed a cheap laser weeding device and experimentally explored the weeding efficiency of 0.3 W, 1 W and 5 W lasers. Experimental results show that 0.3 W, 1 W and 5 W need to irradiate weeds for 76 s, 23 s and 6 s respectively to achieve better weeding effects. Wang M. et al. (2022) proposed a new laser weeding device equipped with a 90 W, 810 nm, 1.8 mm spot diameter laser and a two-degree-of-freedom five-turn rotating parallel manipulator for dynamic intra-row weed removal. Under dynamic conditions, the weeding success rate was 99.2%, the weeding efficiency was 0.73 s/weed, and the residence time was 0.64 s, the weed positioning speed is 0.1 m/s. Zhu et al. (2022) designed a corn seedling field weeding robot based on YOLOX convolutional neural network, verifying the feasibility of blue laser as a non-contact weeding tool. Experimental results showed that the average dry weight prevention effect of the weeding robot was 85%. Yu et al. (2024) conducted an experiment on laser cutting weed stems of four common weeds in farmland (*Chenopodium album*, *Amaranthus spinosus*, *Setaria viridis*, and *Eleusine indica*). The experimental results show that when the irradiation time was 10 s and the irradiation distance was 2 m, the 3.892 W/mm^2^ laser was sufficient to eliminate weeds and plants. When the irradiation distance was 1 m and the irradiation time was within 1 s, the 2.47 W/mm^2^ laser was more effective. Sahin and Cay (2024) used a diode laser with a power of 5500 mW and a wavelength of 450 nm to conduct four irradiation experiments on three weeds (*Galium aparine*, *Scabiosa columbaria*, *Euphorbia helioscopia*), and concluded that laser application to the apical meristem region of weeds may not be effective enough after the cotyledon stage, while application to the plant stems can successfully control these three weeds. Andreasen et al. (2024a) used a 50w thulium-doped fiber laser with a diameter of 2 mm and a wavelength of 2 µm to conduct laser weeding experiments. The experimental results found that when the grass plant (*Alopecurus myosuroides*) has one leaf and the dicotyledonous plant is in the cotyledon stage, the laser irradiation Highest efficacy. In addition, they found that different categories of weeds had different sensitivities to lasers. At the 4-leaf stage, most species would regrow after irradiation. Therefore, irradiating the weeds when they were in the cotyledon to 2-leaf stage could prevent them from regrowth. Christian Andreasen’s team also investigated how to use lasers to kill the widespread and aggressive perennial weed *Elymus repens* after cutting the rhizomes into pieces. The experiments showed that *Elymus repens* plants can be killed using small doses of laser light (less than 1.6 J mm^−2^ in many cases). In general, the best results were achieved when treating small rhizomes at the 3-leaf stage (Andreasen et al., 2024b). Qin et al. (2024) built an intelligent weed detection and laser weeding system and conducted laser weeding experiments. The experimental results showed that laser weeding was feasible at a power of 100 W and a scanning speed of 80 mm/s. The activity of Veronica officinalis was significantly lost within 15 days after weeding, and it did not re-sprout. The laser weeding robot developed by CARBON ROBOTICS is a model of the current commercial application of laser weeding. The latest generation of intelligent laser weeding robots uses a 150 W CO2 laser with a 30 mm level accuracy and can work 24 hours a day. Laser weeding has many advantages, including being environmentally friendly and not damaging the root system of crops. However, the cost of laser is high, and one irradiation can only remove one weed. Therefore, laser weeding can only be applied to fields with only a few weeds in the row, taking cost into account and weeding speed. How to use a smaller number of lasers while ensuring weed control efficiency and quickly removing weeds in high-density intra-rows is the main challenge facing laser weed control.

Currently, the challenges faced by mechanical, chemical and laser weeding cannot be effectively addressed using a single weeding method. Multiple weed control methods in synergy to remove weeds were proposed (Bawden et al., 2017). Fang et al. (2022) explored the possibility of mechanical-chemical synergistic weed control. Field experiments showed that compared with mechanical weed control and chemical weed control, mechanical-chemical synergistic weed control had the best effect, reducing chemical use by 50%. Reducing the amount of chemical application had no significant effect on crop growth and yield. Recently, another study has proved that the combination of mechanical and chemical weeding is more effective than a single mechanical weeding and chemical weeding (Parasca et al., 2024). To solve the difficulties faced by mechanical weeding and laser weeding, this study proposed a mechanical-laser collaborative intra-row weeding method. This method mainly uses mechanical weeding, with laser weeding as an auxiliary. The idea of this weeding method is to use mechanical weeding when the intra-row weed density is high, and laser weeding when the intra-row weed density is low. When there are no weeds in the intra-row, the weeding device does not perform any operation. In addition, weeds adjacent to crops are also removed using lasers. This not only solves the problem that mechanical weeding cannot remove weeds close to crops, but also solves the problem of using a small number of lasers to remove high-density weeds. At the same time, mechanical weeding and laser weeding are environmentally friendly. Currently, there is no intelligent weeding equipment suitable for the mechanical-laser collaborative intra-row weeding method. here are three main challenges in developing a mechanical-laser collaborative intra-row weeding device, including the structural design of the device, the identification and positioning of vegetables-weeds and intra-row weed severity classification, and the development of the control system.

In this study, the structure of a mechanical-laser collaborative intra-row weeding device was proposed for weed control in lettuce fields. To make the design of the device more reasonable, the following research was conducted: (1) A size optimization algorithm for the slider-crank mechanism was proposed. (2) EDEM simulation was used to analyze the stress of weeding knives with different parameters in the soil. (3) The removing capabilities of Near-infrared (NIR) and blue lasers with different powers, Irradiation time and wavelength on weeds were explored. Finally, based on the above research results, a prototype was produced. As far as we know, the intra-row weeding method combining mechanical weeding and laser weeding is a new method, and the weeding device suitable for the weeding method proposed in this article is also novel.

## 2 Materials and methods

### 2.1 The overall structure of the new weeding device

**Figure 1:**
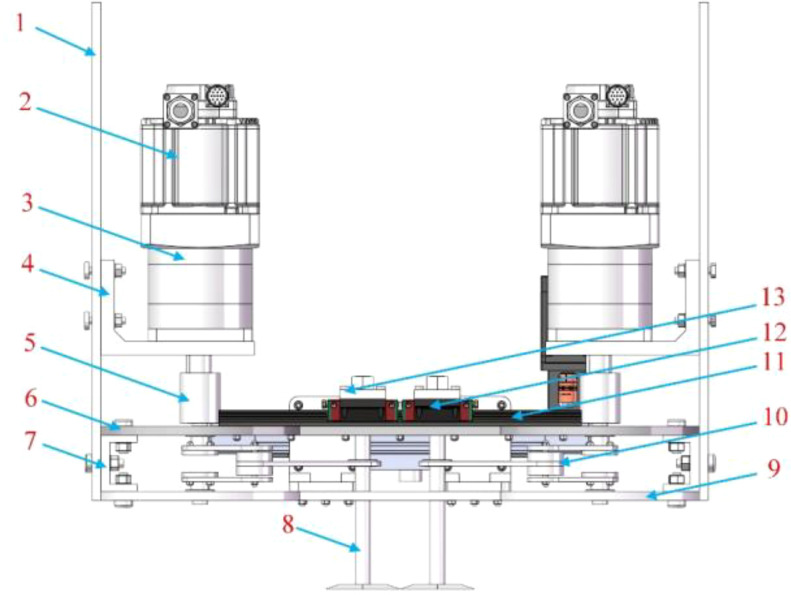
Front view of a 3D model of the mechanical-laser collaborative intra-row weeding device. 1-Weeding device-vehicle platform connector, 2-Motor I, 3-Reducer, 4-Motor support, 5-Coupler, 6-Upper load-bearing plate, 7-Load-bearing plate connector, 8-Weeding knife, 9-Lower load-bearing plate, 10-Slider-crank mechanism, 11- Guide rail, 12-Slide block, 13-Weeding knife-sliding block connector.

**Figure 2:**
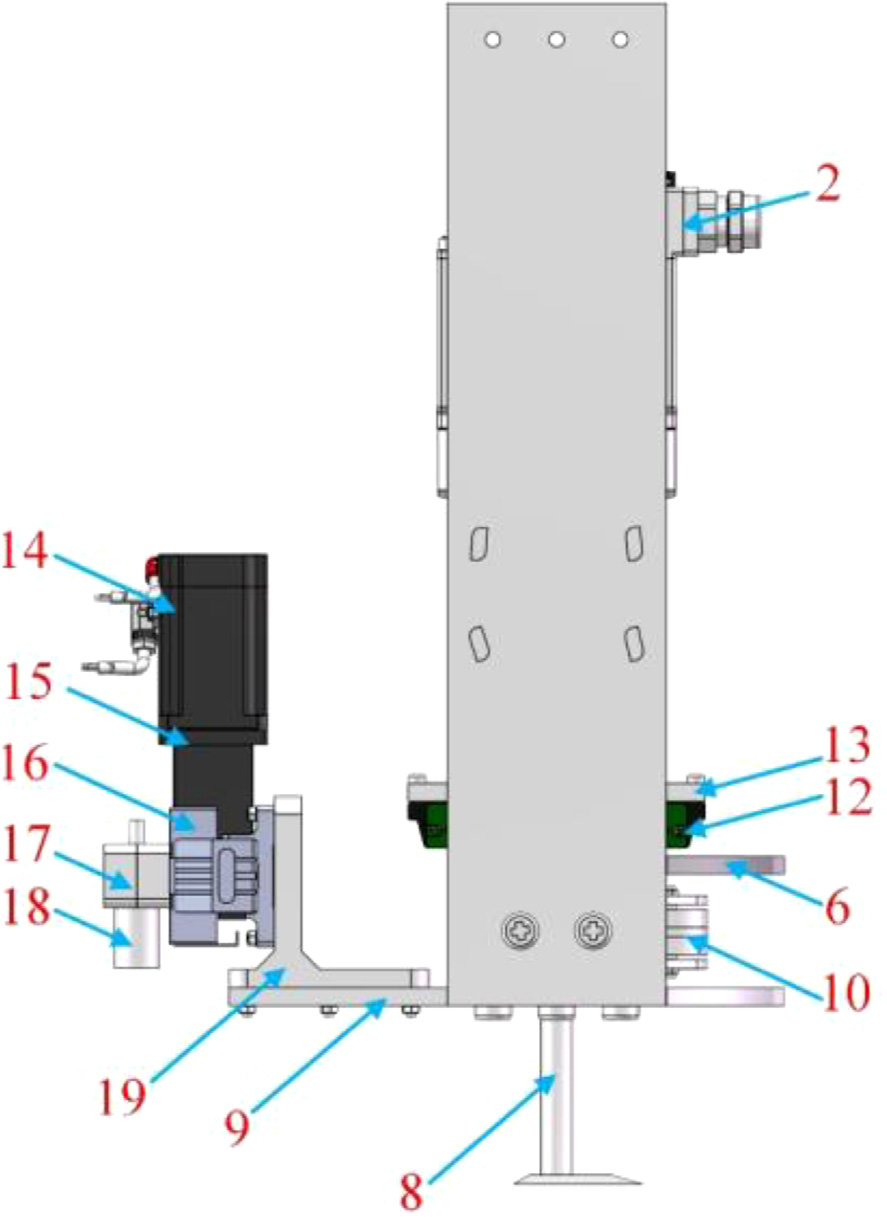
Side view of a 3D model of the mechanical-laser collaborative intra-row weeding device. 14-Motor II, 15- Motor II support, 16-Laser head fixture, 17-Synchronous belt, 18-Laser head, 19-Synchronous belt-lower load-bearing plate connector.

**Figure 3:**
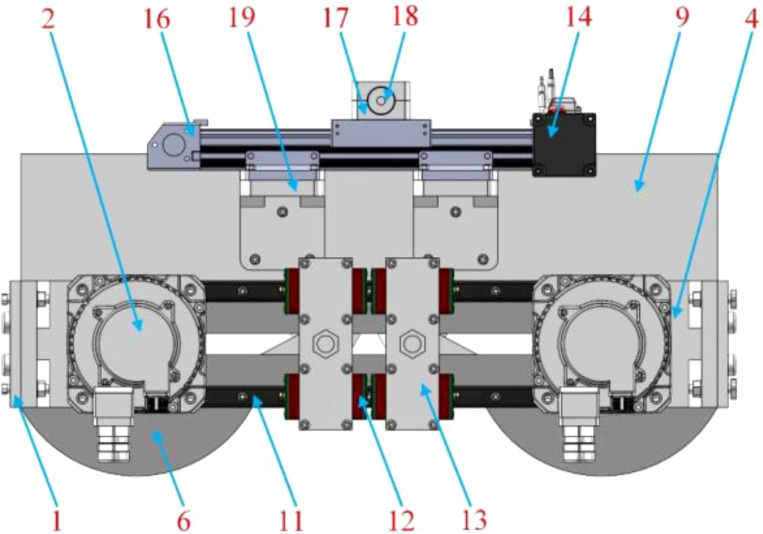
Top view of a 3D model of the mechanical-laser collaborative intra-row weeding device.

The mechanical-laser collaborative weeding method is a new weeding method. There is currently no weeding robot that uses this weeding method. Therefore, this study proposed a novel mechanical-laser collaborative intra-row weeding device for intra-row weed removal in vegetable fields. The front view, side view and top view of the 3D model of the weeding device are shown in **Figures 1**-**3** respectively. The weeding device was divided into a mechanical weeding part and a laser weeding part. The structure of the mechanical weeding part is shown in **Figures 1**, **3**. It was mainly composed of motor, slider-crack mechanism, slider block, guide rail and weeding knife. The motor reduced the speed and increased the torque through the reducer. Then, the motor and reducer were connected with the slider-crank mechanism through a coupling. Finally, the slider-crank mechanism was connected to the weeding knife. In this way, the rotational motion of the motor can be converted into linear motion through the slider-crank mechanism, thereby controlling the motion of the weeding knife. To make the movement of the weeding knife more precise, the weeding knife and the guide rail slider mechanism were connected through the weeding knife-sliding block connector. The structure of the laser weeding part are shown in **Figures 2** and **3**. The laser weeding part mainly consisted of laser head, synchronous belt, motor, and laser head fixture. The motor and the synchronous belt were connected through the motor support II and coupling. The laser head was connected with the synchronous belt through the laser head fixture. In this way, the rotational motion of the motor was converted into the linear motion of the laser head through the synchronous belt. The laser weeding part was connected to the lower load-bearing plate through the Synchronous belt-lower load-bearing plate connector. The upper load-bearing plate and the lower load-bearing plate were connected through a load-bearing plate connector, and the mechanical weeding part is fixed. The entire weeding device was connected to the mobile robot platform via the weeding device-vehicle platform connector.

### 2.2 Design and analysis of slider-crank mechanism

#### 2.2.1 Dimensional optimization of slider-crank mechanism

The slider-crank mechanism can convert the rotary motion of a motor into a linear motion and widely used in a variety of devices, such as internal combustion engines, presses and high-speed printing presses. The performance of a slider-crank mechanism is affected by many factors, including size, transmission angle, and working space. In this section, an optimization algorithm for the slider-crank mechanism will be proposed, which comprehensively considers the crank length, link length, transmission angle and working space.

**Figure 4:**
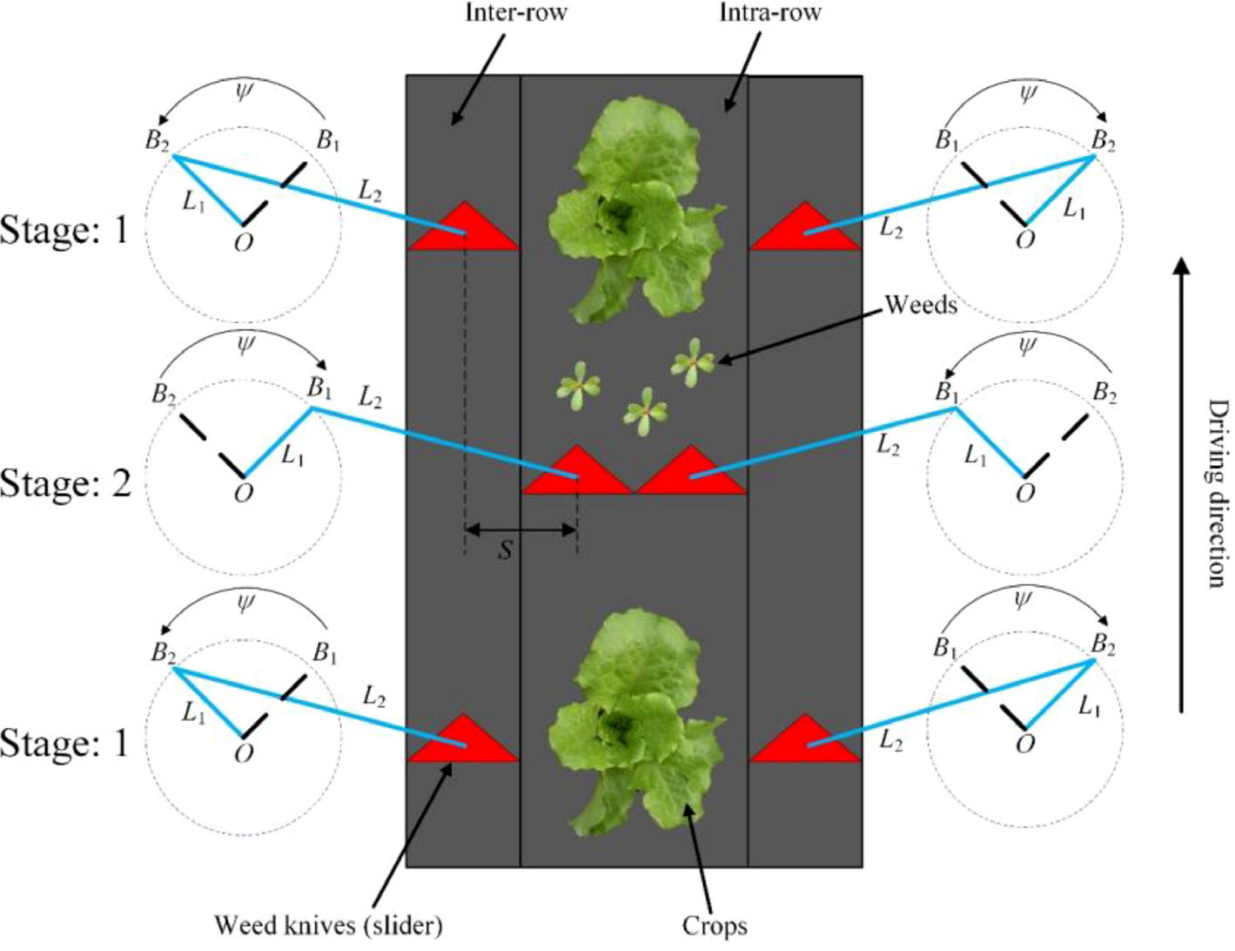
The principle of weeding knife avoiding seedlings under the control of crank slider mechanism.

**Figure 4** shows the method of the slider-crank mechanism to control the weeding knife to avoid crops. In **Figure 4**, *L*_1_ represents the crank length, *L*_2_ represents the link length, the slider-crank mechanism rotates along the point *O*, *ψ* represents the rotating range of the crank, and *S* represents the working range of the weed knife (slider). In stage 1, the crank of the slider-crank mechanism rotates from *B*_1_ to *B*_2_ under the drive of the motor, and the weed knife move into inter-row. In stage 2, the crank of the slider-crank mechanism rotates from *B*_2_ to *B*_1_ under the drive of the motor, and the weeding knife moves into intra-row.

**Figure 5:**
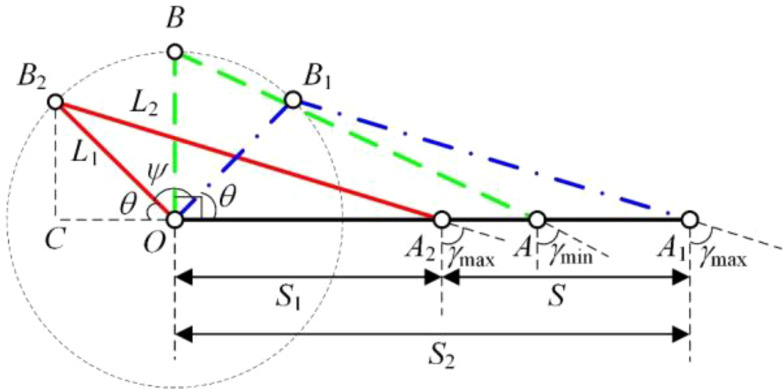
Solution model of slider-crank mechanism optimization.

**Figure 5** shows the equivalent model of the slider-crank mechanism. *γ* denotes the transmission angle of the slider-crank mechanism, *S*_1_ denotes the shortest distance of the weed knife (slider) from point *O*, and *S*_2_ denotes the longest distance of the weed knife (slider) from point *O*. During the motion of the slider-crank mechanism, the transmission angle *γ* varies periodically. Therefore, it is assumed that the range of values for the minimum transmission angle (*γ*_min_) is [*γ*_min1_, *γ*_min2_] and the range of values for the maximum transmission angle (*γ*_max_) is [*γ*_max1_, *γ*_max2_].

In the right triangle *OBA*, *γ*_min_ is related to *L*_1_ and *L*_2_ as shown in Equation 1.


(1) $$\cos\left(\gamma_{\text{min}}\right)=\frac{L_{1}}{L_{2}}$$

In the right triangle *CA*_2_ *B*_2_, *γ*_max_ is related to *θ*, *L*_1_ and *L*_2_ as shown in Equation 2.


(2) $$\cos\left(\gamma_{\text{max}}\right)=\frac{L_{1}\times \sin\theta }{L_{2}}$$

The Equation 3 can be obtained from Equations 1 and 2.


(3) $$\cos\left(\gamma_{\text{max}}\right)=\cos\left(\gamma_{\text{min}}\right)\times \sin\theta$$

Therefore, the range of values of *θ* is [ $\sin^{-1}\left(\frac{\cos\gamma_{\text{max}2}}{\cos\gamma_{\text{min}}}\right),\;\sin^{-1}\left(\frac{\cos\gamma_{\text{max}1}}{\cos\gamma_{\text{min}}}\right)$ ].

In triangle *CA*_2_ *B*_2_, *S*_1_ is related to *θ*, *L*_1_ and *L*_2_ as shown in Equation 4.


(4) $$S_{1}=L_{2}\times \cos\left(90^{{}^{\circ}}-\gamma_{\text{max}}\right)-L_{1}\times \cos\theta$$

Similarly, *S*_2_ is related to *θ*, *L*_1_ and *L*_2_ as shown in Equation 5.


(5) $$S_{2}=L_{2}\times \cos\left(90^{{}^{\circ}}-\gamma_{\text{max}}\right)+L_{1}\times \cos\theta$$

Thus, *S* can be expressed by Equation 6.


(6) $$S=S_{2}-S_{1}=2\times L_{1}\times \cos\theta$$

Ultimately, the relationship of *L*_1_ with *θ* and *S* can be expressed by Equation 7.


(7) $$L_{1}=\frac{S}{2\times \cos\theta }$$

#### 2.2.2 Design of optimization algorithms

In Section 2.1.1, the relationships between some of the parameters of the slider-crank mechanism were derived. In this section, these parametric relationships were used to design the sizing optimization algorithm for the slider-crank mechanism. The algorithm was developed in MATLAB software. The optimization process for sizing the slider-crank mechanism is described in **Algorithm 1**. Where *S*, *γ*_min1_, *γ*_max1_, *γ*_min2_, *γ*_max2_, *γ*_min_, *L*_1_, *L*_2_ have the same meaning as in Section 2.1.1. *γ*_mint_ represents the minimum transmission angle of the mechanism after the values of *L*_1_ and *L*_2_ have been determined. The parameters *γ*_maxt_ and *θ*_truth_ have a meaning like that of *γ*_mint_. Considering issues such as row spacing in lettuce planting, the initial input parameters *γ*_min1_, *γ*_min2_, *γ*_min_, *γ*_max1_, *γ*_max2_, and *S* were set to 49°, 51°, 50°, 60°, 70°, and 120 mm, respectively.

Algorithm 1 **Optimization of slider-crank mechanism dimensions.**

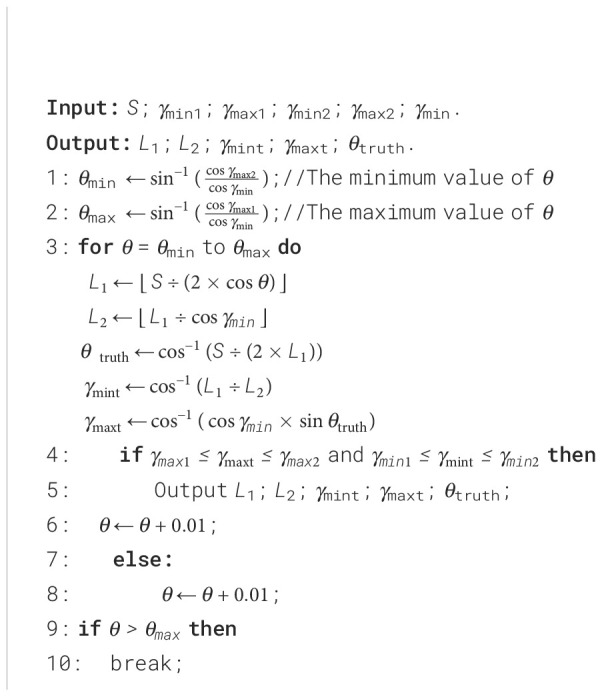

#### 2.2.3 Verification of dimensional optimization results

ADAMS is a multi-rigid body dynamics software. Users can use the software to analyze the statics, kinematics, and dynamics of virtual mechanical systems very easily. In this section, ADAMS software was used to validate the correctness of the slider-crank mechanism optimization algorithm for dimensioning proposed in Sections 2.2.1 and 2.2.2. First, the lengths of the crank and link were determined based on the initial input parameters using the proposed **Algorithm 1**. Then, a 3D model of the slider-crank mechanism was established in SOLIDWORKS and saved in .x_t format. Finally, the .x_t file imported into the ADAMS software and set constraints, drive parameters, and measurements. The simulation time was 1 s. The crank rotation speed was (180-2×*θ*_truth_) deg/s. The direction of motion was clockwise.

### 2.3 Weeding knife simulation experiment

Weeding knife is a key part of mechanical weeding robots. Weeding knives with different parameters are subjected to different forces in the soil. The resistance of the weeding knife further affects the parameters of the motor. Therefore, the lower the resistance of the weeding knife, the smaller and less costly the weeding device will be. In this section, the triangular weeding knife was used as a prototype, and the resistance of weeding knives with different penetration angles and edge angles under the same simulation environment was simulated and analyzed in EDEM software. Finally, the parameters of the weeding knife were determined based on the simulation results. EDEM is a specialized software for simulating the behavior of granular materials and is widely used to simulate cases of force analysis of farming tools in mutual contact with soil (Aikins et al., 2021b; Liu et al., 2023; Wang et al., 2019, Wang X. et al., 2022). The accuracy of the simulation results is related to the soil model, the farming tools-soil contact model, and the soil-soil contact model. Therefore, the parameter settings in the relevant literature published in recent years were summarized as shown in **Table 1**. The two main contact modeling options in published articles were Hysteretic Spring + Linear Cohesion (Aikins et al., 2021a, 2021b, 2021c; Awuah et al., 2022; Makange et al., 2020; Saunders et al., 2021; Shi et al., 2019; Wang et al., 2020) and Hertz-Mindlin + bonding (or bonding V2) (Liu et al., 2023; Wang X. et al., 2019, Wang Y. et al., 2019; Zhang L. et al., 2023, Zhang P. et al., 2023). In addition, there were some articles using both EEPA (Kim et al., 2021; Sun et al., 2020) and Hertz-Mindlin with JKR + bonding (bonding V2) (Song et al., 2022; Zhang et al., 2022; Zhao et al., 2023) as contact models.

**Table 1:** Summary of EDEM properties.

Property	Value
Poisson’s Ratio	0.3 - 0.4
Solids Density (kg/m^3^)	1130 - 2680
Shear Modulus (Pa)	1 × 10^6^ - 1.02 × 10^8^
Young’s Modulus (Pa)	5.2 × 10^7^ - 1.8 × 10^8^
Particle physical Radius (mm)	3 - 10
Coefficient of restitution (soil - soil)	0.2 - 0.6
Coefficient of static friction (soil - soil)	0.107 - 0.775
Coefficient of rolling friction (soil - soil)	0.02 - 0.6
Coefficient of restitution (soil - tool)	0.05-0.6
Coefficient of static friction (soil - tool)	0.24-0.85
Coefficient of rolling friction (soil - tool)	0.05-0.6

In summary, the soil property settings for the simulation experiment in this section are shown in **Table 2**. The contact model was set to Hysteretic Spring + Linear Cohesion V2 (https://2022.help.altair.com/2022.1/EDEM/Creator/Physics/Additional_Models/Linear_Cohesion_V2.htm). Linear Cohesion V2 was an improvement on the Linear Cohesion model as it was better for non-uniform particle size distributions. More importantly, Linear Cohesion V2 can use GPU, which greatly reduces simulation time. The main configuration of the computer used for simulation is AMD Ryzen 5 5600X 6-core processor and NVIDIA RTX 3080Ti.

**Table 2:** Soil particle properties in EDEM simulation.

Property	Value
Poisson’s Ratio	0.3
Solids Density (kg/m^3^)	2000
Young’s Modulus (Pa)	1.8 × 10^8^
Particle physical Radius (mm)	0.9 - 1.5
Particle size distribution	random
Coefficient of restitution	0.3
Coefficient of static friction	0.36
Coefficient of rolling friction	0.18

The soil-to-soil and soil-to-weeding knife contact model properties were set as described in **Table 3**.

**Table 3:** Property values for soil-to-soil and soil-to-weeding knife contact modeling.

Property	Value
Particle to Particle (base)	Hysteretic Spring
Particle to Particle (additional)	Linear Cohesion V2
Damping factor	0.05
Stiffness factor	0.85
Yield Strengths (Pa)	2.8 × 10^6^
Energy Density (J/m^3^)	46400
Particle to Geometry (base)	Hysteretic Spring
Particle to Geometry (additional)	Linear Cohesion V2
Damping factor	0.05
Stiffness factor	0.85
Yield Strengths (Pa)	3.8 × 10^8^
Energy Density (J/m^3^)	14900

The properties of the weeding knife in EDEM were set as described in **Table 4**.

**Table 4:** Weeding knife properties in EDEM simulation.

Property	Value
Poisson’s Ratio	0.29
Solids Density (kg/m^3^)	7801
Shear Modulus (Pa)	8.023× 10^10^
Coefficient of restitution	0.6
Coefficient of static friction	0.712
Coefficient of rolling friction	0.216

The design of the simulation experiment with edge angle and penetration angle of the weeding knife as variables is shown in **Table 5**. Test 1 shows that the blade angle and penetration angle of the weeding knife are 30° and 0° respectively, and the others are similar. The weeding knife is an equilateral triangle with a side length of 75 mm and a thickness of 5 mm.

**Table 5:** Results of the design of simulation experiments for weeding knife.

Penetration angle Edge angle	0°	1°	2°	3°	4°	5°
30°	Test 1	Test 2	Test 3	Test 4	Test 5	Test 6
45°	Test 7	Test 8	Test 9	Test 10	Test 11	Test 12
60°	Test 13	Test 14	Test 15	Test 16	Test 17	Test 18

**Figure 6:**
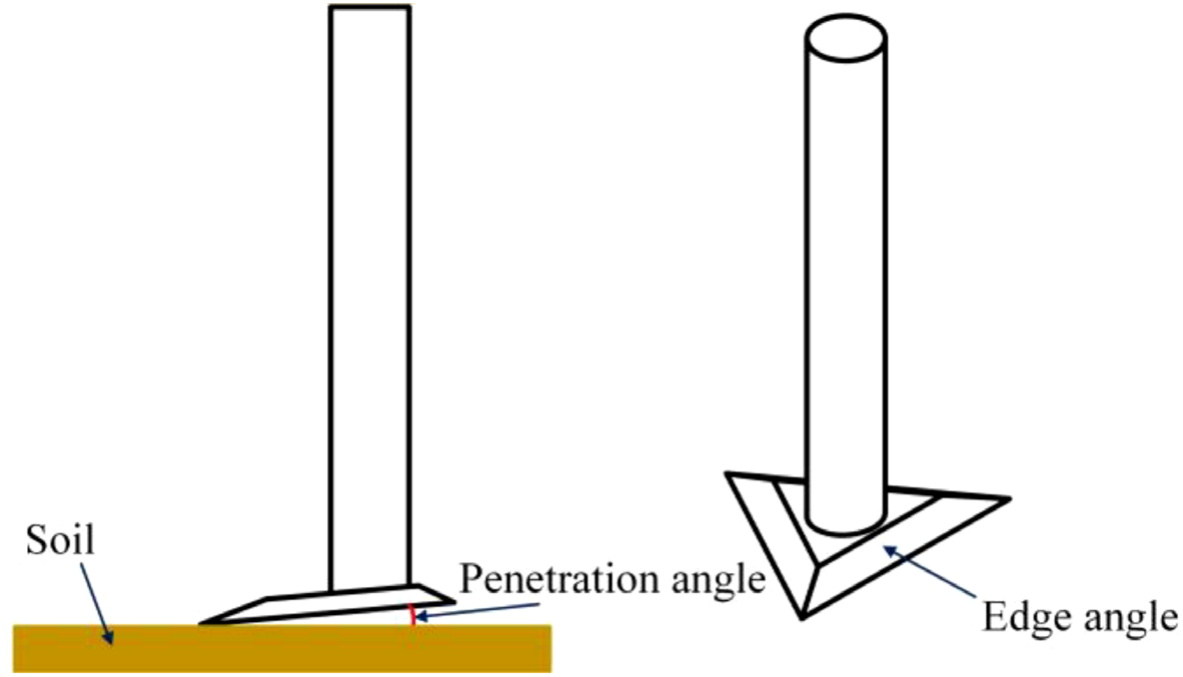
Explanation of the edge angle and penetration angle of the weeding knife.

The edge angle and penetration angle of the weeding knife are shown in **Figure 6**.

**Figure 7:**
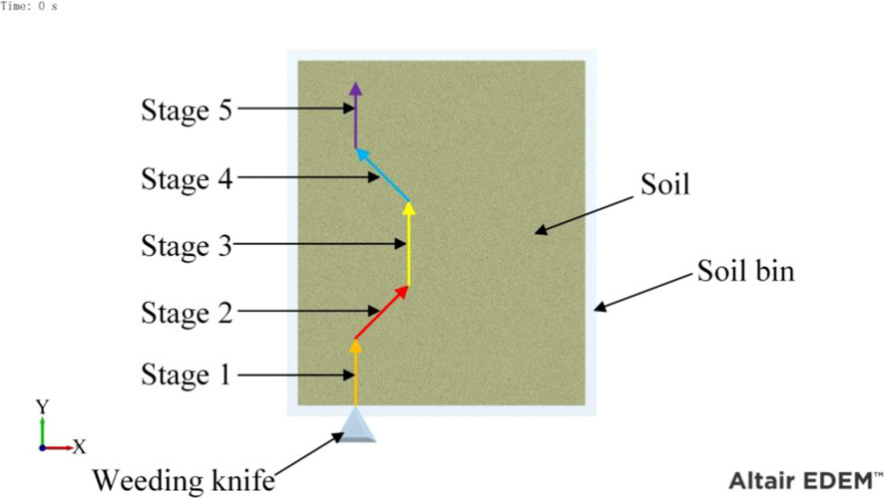
Simulated motion path of the weeding knife.

As shown in **Figure 7**, the soil particles were filled in a soil bin. The length, width and depth of the soil bin were 600 mm, 500 mm and 100 mm respectively. The depth of the weeding knife into the soil was 10 mm, and the simulation time was 0.5 s. The path of movement of the weeding knife Consisted of stages 1 to 5. The time of each stage was 0.1 s. Stages 1 and 5 simulated the movement of the weeding knife in the inter-row with a forward speed of 1 m/s. Stage 2 simulated the weeding knife entering intra-row, moving forward at 1 m/s and moving to the right at 1 m/s. Stage 3 simulated the movement of the weeding knife in the intra-row at a speed of 1 m/s. Stage 4 simulated the weeding knife entering inter-row, moving forward at 1 m/s and moving to the left at 1 m/s.

### 2.4 Laser weed control experiment

As researchers delve deeper into laser weed control, it has been proven to be a very promising intelligent weed control technology. However, there are still many issues that need to be studied urgently regarding the effect of laser parameters on weed control, such as wavelength and irradiation time, and the types of weeds. For example, different types of weeds have different sensitivities (absorption rates) to different wavelengths. Larger weeds require more energy to be killed. The energy generated by the laser is related to the laser power and light emission time.

**Figure 8:**
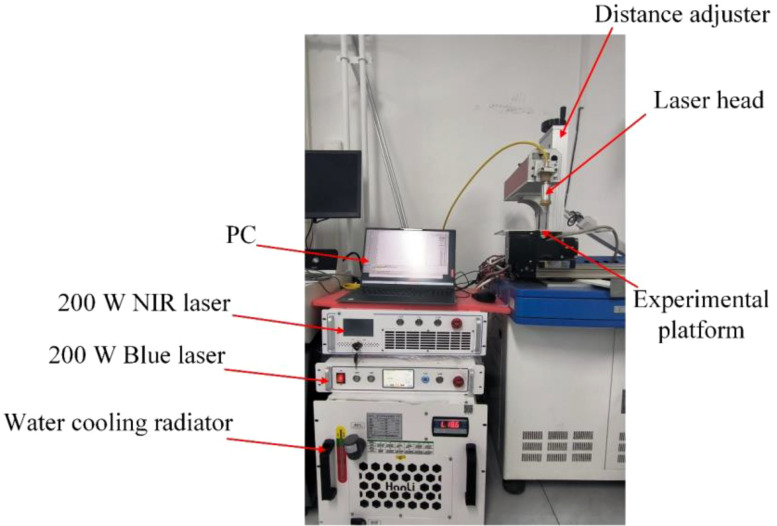
Description of the laser weeding experimental site and equipment.

In this section, 200 W NIR and 200 W blue lasers (HAN’S TCS, Beijing, China) with adjustable power and irradiation time were used to explore the effects of wavelength, power, and irradiation time on weeding efficiency. The parameters of the lasers used in the experiment are described in **Table 6**. The experimental site is shown in **Figure 8**. The weeds used in the laser experiment came from the vegetable greenhouse in Haidian, Beijing. To make the experimental results as accurate as possible, the weed seedlings (2 - 4 leaves) used to explore the wavelength, power and irradiation time were all *Portulaca oleracea L.* There is no strict requirement for the species of large weeds to be consistent, in the hope that the experimental results will be more applicable. These weeds were collected from the greenhouse the day before the experiment and placed in culture trays to ensure the activity of the weeds during the experiment the next day. For the experiment, the laser spot diameter is controlled at about 1 mm and the laser was stationary and pointed vertically downward at the center of the weed stem. Laser weed control is considered successful when the leaves of the weeds are knocked off or the stems are burned. By adjusting the power of the laser output, 200 W, 100 W, 75 W and 50 W NIR laser weeding experiments were carried out. In addition, 100 W, 50 W blue laser weed control experiments (2 - 4 leaves). Not all weeds in the field are in the seedling stage. Therefore, after the laser parameters were initially determined, experiments were also conducted to control larger weeds with laser irradiation. Each set of experiments was performed three times to prevent chance events. The experiment was successful only if all three experiments removed the weeds.

**Table 6:** Basic parameters of the two lasers.

Laser type Parameters	NIR	Blue
CW output power (W)	200	200
Center wavelength (nm)	915 ± 10 980 ± 10	450 ± 10
Spectral width (90% of Power) (nm)	< 10.0	4.0
Power adjustment range (W)	0 - 200	0 - 200
Spot size (mm)	1	1
Output method	Continuous output	Continuous output
Control method	Local control	RS232
Cooling method	Water cooling (25°C)	Water cooling (25°C)

## 3 Results

### 3.1 Dimensional optimization and simulation verification of slider-crank mechanism

**Figure 9:**
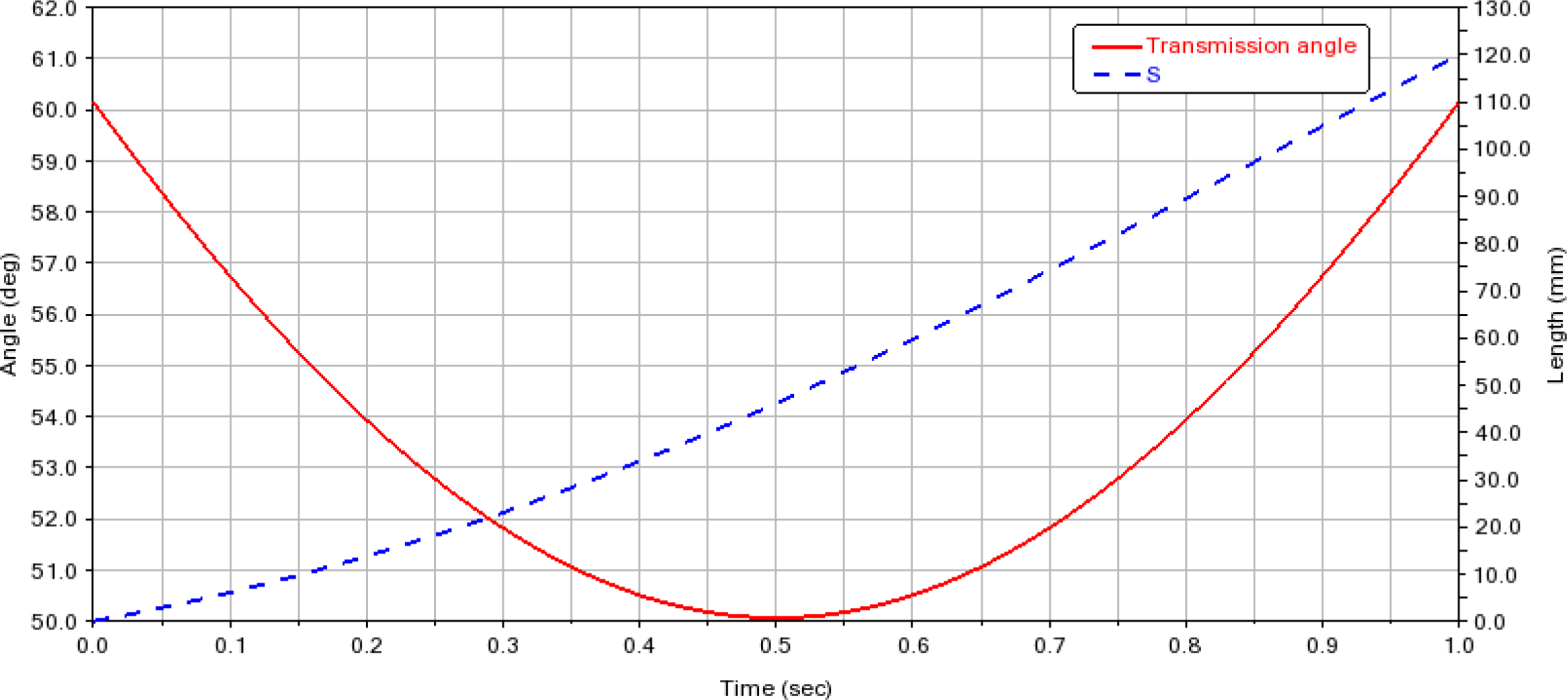
Kinematic simulation results of the slider-crank mechanism.

To obtain the optimized dimensions of the slider-crank mechanism and verify the correctness of the algorithm. Input the initial input parameters into the algorithm and run the code to get *L*_1_ = 87 mm, *L*_2_ = 135 mm, *θ*_truth_ = 46.397187°, *γ*_mint_ = 49.875965°, *γ*_maxt_ = 60.717813°. **Figure 9** is the kinematic simulation result of the slider-crank mechanism. The red solid line in the figure represents the real-time distance change of the slider (weeding knife), and the blue dotted line represents the real-time transmission angle change of the crank slider mechanism. The simulation results showed that *γ*_mint_ = 50.067°, *γ*_maxt_ = 60.1558°, and *S* = 120.0085 mm. The simulation results were basically consistent with the theoretical calculation results.

### 3.2 Results of EDEM simulation experiments

Before determining the soil penetration angle and edge angle parameters of the weeding knife, a simulation experiment was carried out using EDEM simulation software. During the simulation time of 0.5 s, the EDEM software recorded 1000 data points. The original data results of simulating the resistance of the weeding knife in the soil in EDEM software are shown in **Figuress 10**, **11**. During the simulation process, the changing trends of the resistance of the weeding knife with different parameters were consistent. Among the 5 stages, the resistance of the weeding knife in the 1st (0 - 0.1 s), 3rd (0.2 - 0.3 s) and 5th (0.4 - 0.5 s) stages was smaller than that in the 2nd (0.1 - 0.2 s) and 4th (0.3 - 0.4 s) stages. In the 1st, 3rd and 5th stages, the weeding knife only moved inter-row or intra-row. The total resistance of the weeding knife varies in the range of 0 - 2 N, and the resistance in the X direction varies around 0 N. In the 2nd and 4th stages, the weeding knife moved in the X direction to avoid the crop seedlings. Therefore, the weeding knife was subject to resistance in two directions. The total resistance varies between 2 - 4 N, and the resistance in the X direction absolute varies between 1 - 6 N. The positive or negative value of the resistance in the X direction represents the direction of the force, not the magnitude. The weeding knife has the greatest resistance at 0.1 s and 0.3 s. At 0.1 s, the weeding knife began to move from inter-row to intra-row. At 0.3 s, the direction of movement of the weeding knife was opposite to that at 0.1 s. In general, the simulation results were credible and consistent with the actual situation.

**Figure 10:**
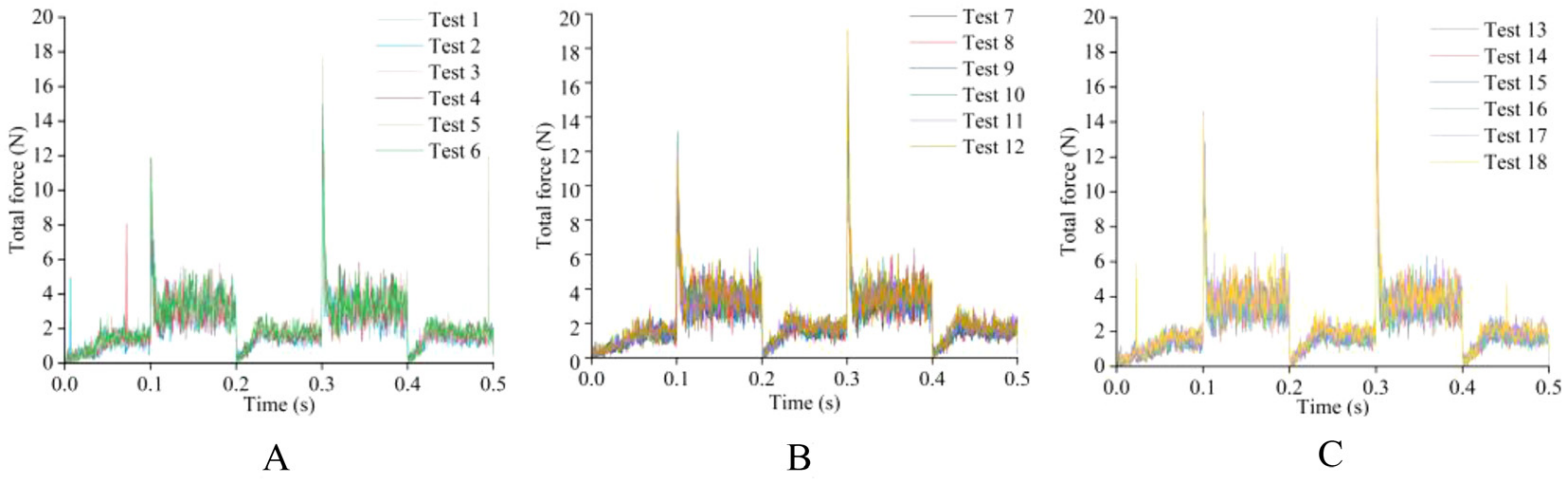
The original data plot of the total resistance of the soil to the weeding knife during the simulation experiment. **(A)** The edge angle is 30° and the angle of penetration is 0° - 5°. **(B)** The edge angle is 45° and the angle of penetration is 0° - 5°. **(C)** The edge angle is 45° and the angle of penetration is 0° - 5°.

**Figure 11:**
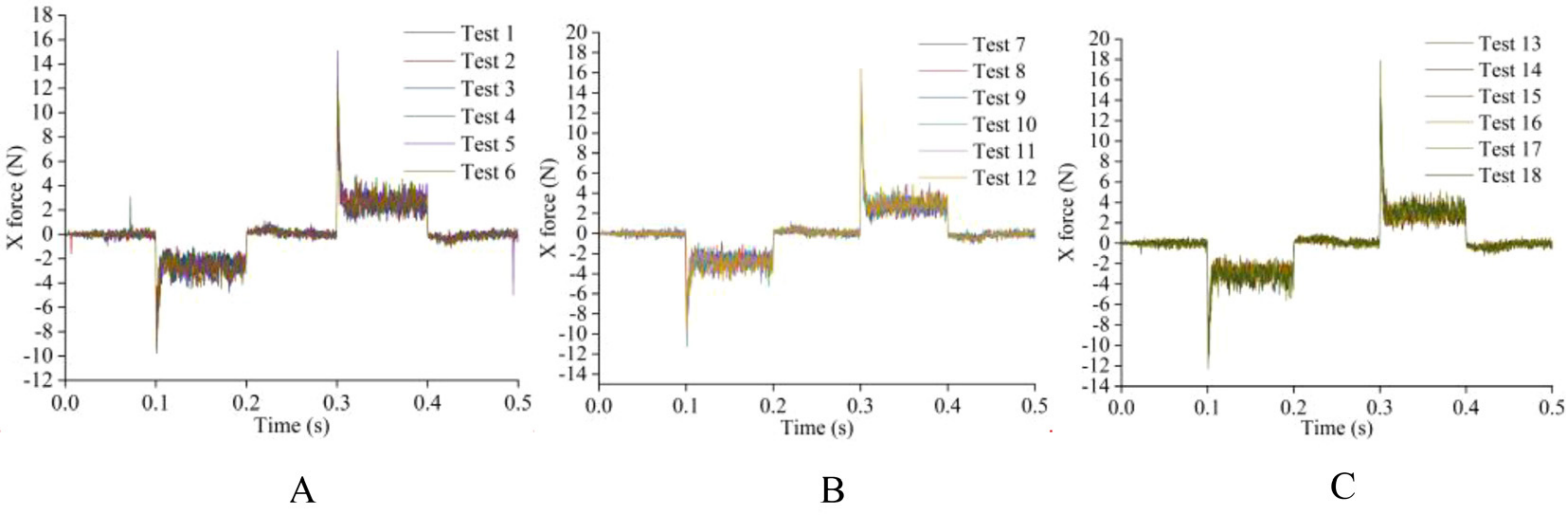
The original data plot of the X direction resistance of the soil to the weeding knife during the simulation experiment. **(A)** The edge angle is 30° and the angle of penetration is 0° - 5°. **(B)** The edge angle is 45° and the angle of penetration is 0° - 5°. **(C)** The edge angle is 45° and the angle of penetration is 0° - 5°.

**Figure 12:**
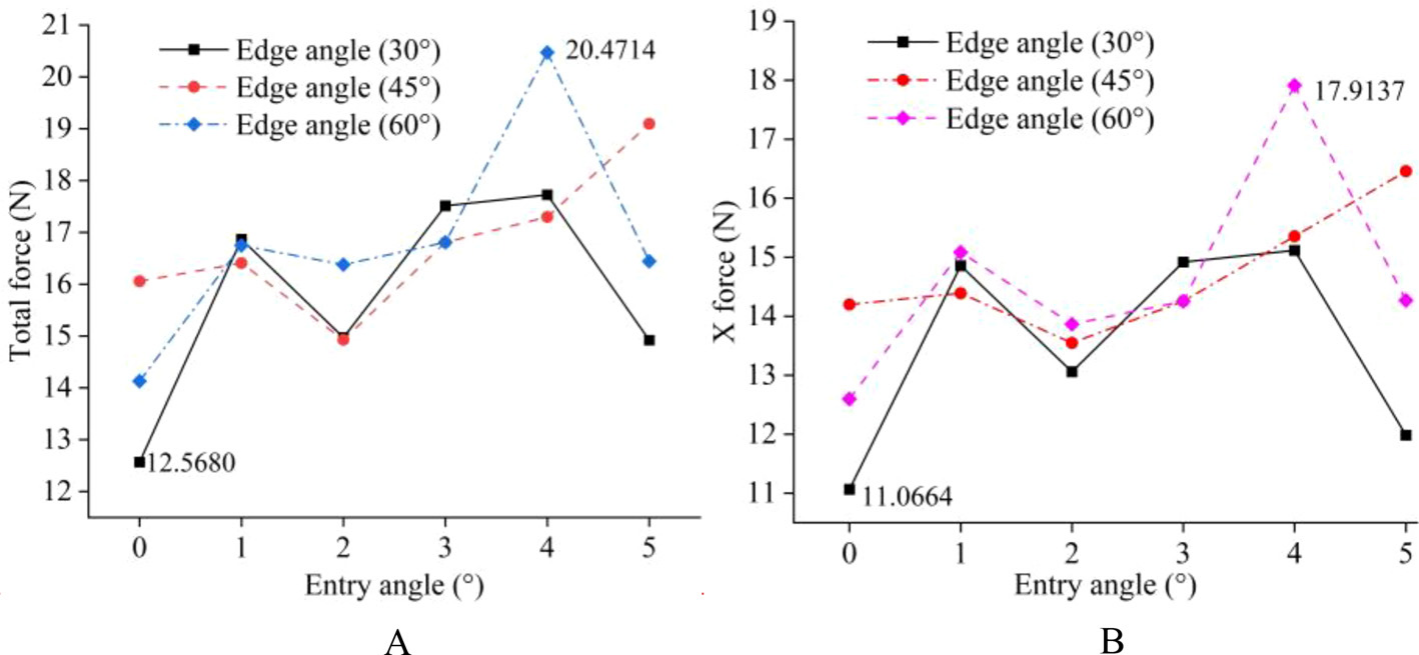
Summary plot of the maximum resistance suffered by weeding knives with different parameters in the simulation experiments. **(A)** Total resistance. **(B)** X-direction resistance.

To more clearly compare the resistance of weeding knives with different parameters, the maximum value of each simulation result was extracted, as shown in **Figure 12**. The total and X directional resistance of the weeding knife at a soil penetration angle of 4° and an edge angle of 60° were the largest among all simulation experiments at 20.4714 N and 17.9137 N, respectively. The total and X directional resistance of the weeding knife when the angle of penetration was 0° and the edge angle was 30° were the smallest in all experiments at 12.5680 N and 11.0664 N, respectively. Therefore, based on the simulation results, the weeding knife of the weeding device designed in this research has a soil penetration angle of 0° and an edge angle of 30°.

### 3.3 Results of laser weed control experiments

**Figure 13:**
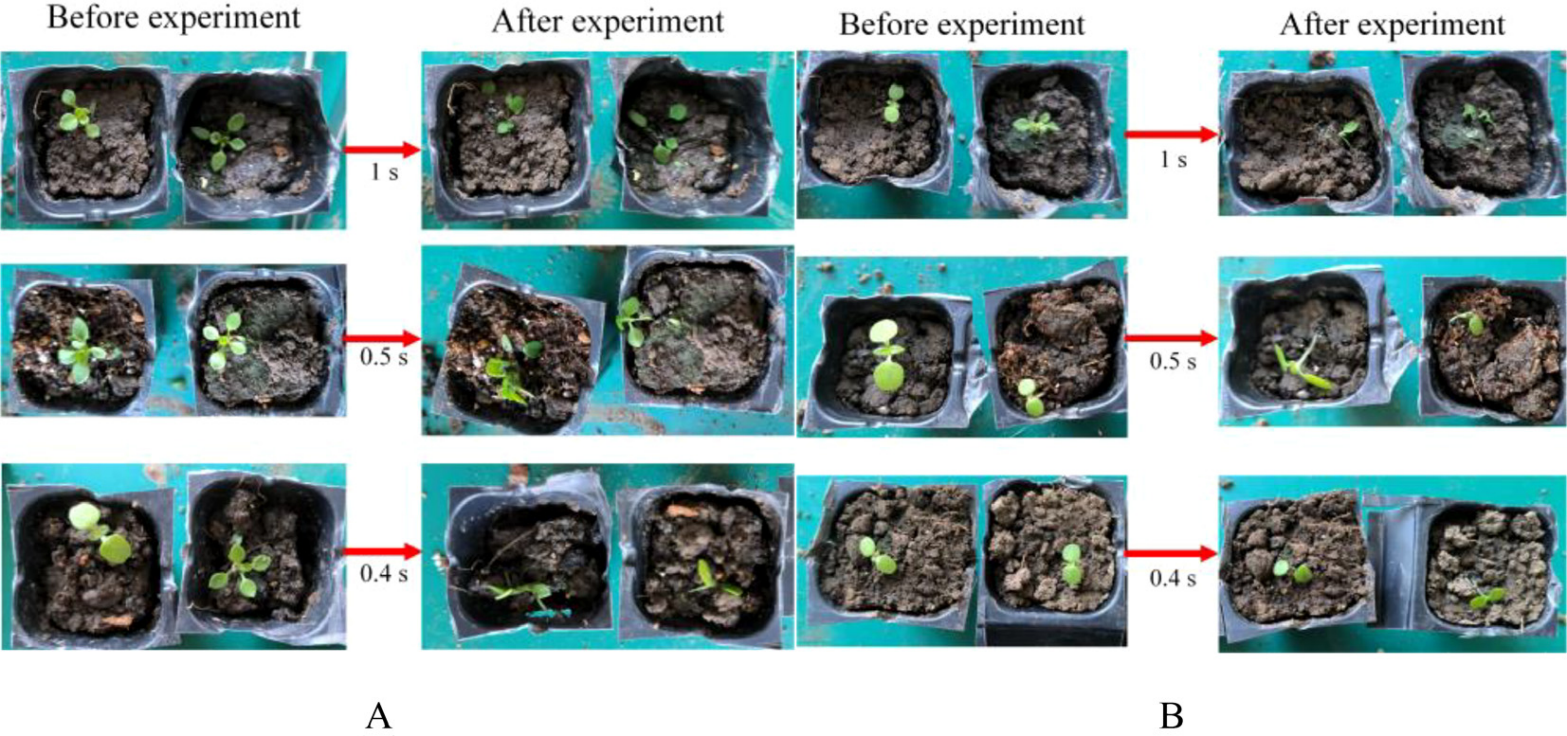
Diagram of the results of a laser weed control experiment. **(A)** 200 W NIR laser weeding experiment, **(B)** 100 W NIR laser weeding experiment.

To determine the power and type of laser in the mechanical-laser cooperative intra-row weeding device, a laser weeding experiment was carried out. The experimental results of the NIR laser irradiation of weeds are shown in **Table 7**. When the power was 200 W, the irradiation time of 1 s, 0.5 s and 0.4 s successfully removed the weeds in all three experiments. When the irradiation time was 0.25 s, only one of the three experiments succeeded in removing the weed. Therefore, it can be inferred that the minimum time for a 200 W NIR laser to remove a weed was between 0.25 and 0.4 s. When the power was 100 W, the three groups of experiments with irradiation times of 1 s, 0.5 s and 0.4 s successfully removed the weeds. At irradiation times of 0.25 s and 0.3 s, all three experiments succeeded only once. Therefore, it can be roughly inferred that the minimum time for a 100 W NIR laser to remove a weed was between 0.3 s and 0.4 s. When the power was 75 W and 50 W, irradiating the weeds for 1 s succeeded only once in all three experiments. The NIR lasers with two powers, 75 W and 50 W, will remove weeds in more than 1 s, and will no longer be suitable for real-time laser weeding robots. Therefore, no further experiments were done. **Figure 13A** shows some experimental results of removing weeds after irradiating weeds with a 200 W NIR laser for 1 s, 0.5 s, and 0.4 s, respectively. **Figure 13B** shows some experimental results of removing weeds after irradiating weeds with a 100 W NIR laser for 1 s, 0.5 s, and 0.4 s, respectively.

**Table 7:** Experimental results of weed control with NIR lasers of different powers.

Power (W)	Irradiation time (s)	Test 1	Test 2	Test 3	Success rate (%)
200	1	✓	✓	✓	100
	0.5	✓	✓	✓	100
	0.4	✓	✓	✓	100
	0.25	✓	✕	✕	33.3
100	1	✓	✓	✓	100
	0.5	✓	✓	✓	100
	0.4	✓	✓	✓	100
	0.3	✕	✕	✓	33.3
	0.25	✓	✕	✕	33.3
75	1	✓	✕	✕	33.3
50	1	✓	✕	✕	33.3

✓ indicates that the experiment is successful, and × indicates that the experiment is failed.

**Figure 14:**
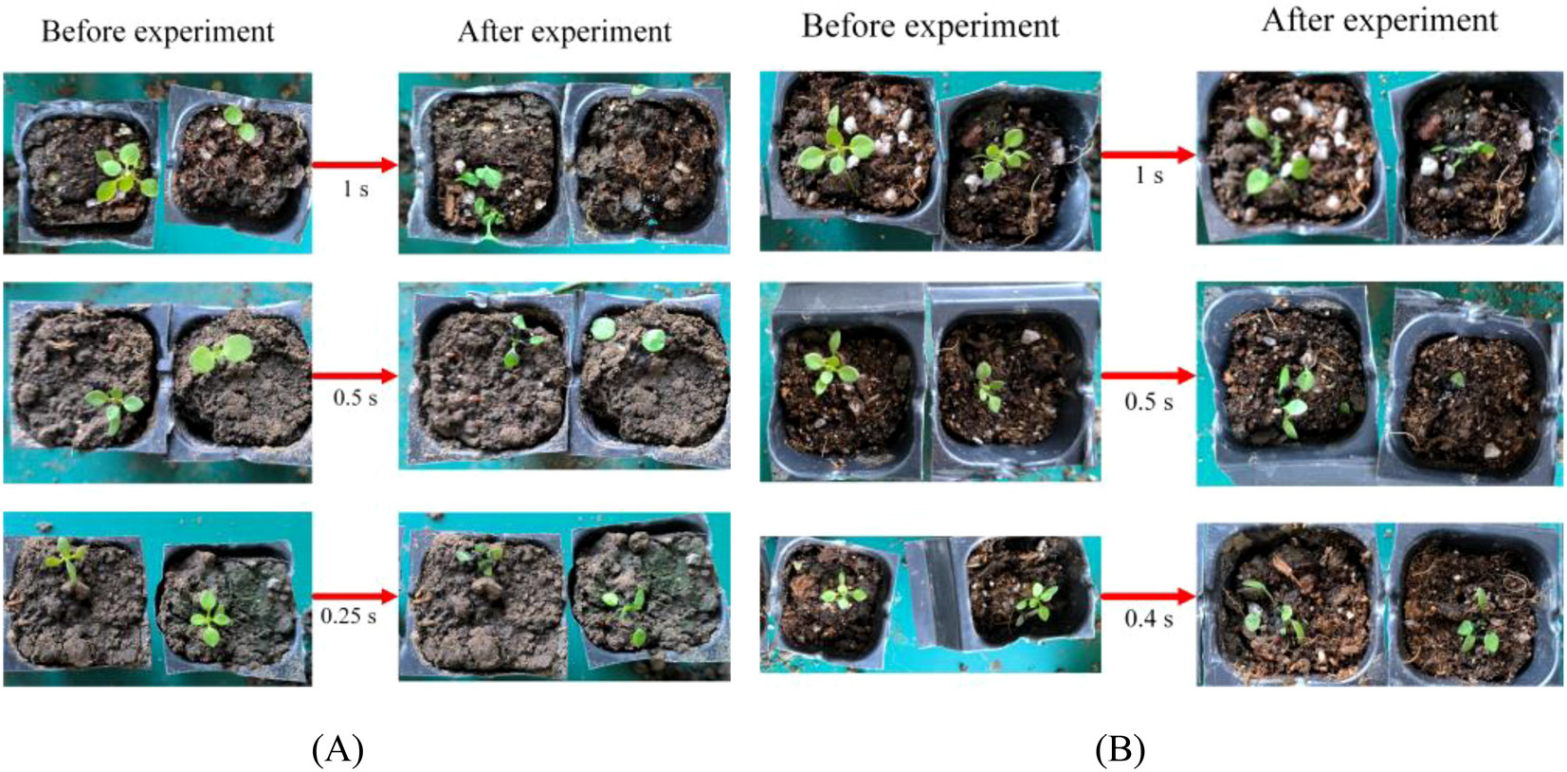
Diagram of the results of a laser weed control experiment. **(A)** 100 W blue laser weeding experiment, **(B)** 50 W blue laser weeding experiment.

The experimental results of the blue laser irradiation of weeds are shown in **Table 8**. When the power was 100 W, the laser irradiated the weeds for 1 s, 0.5 s, and 0.25 s, respectively, and the weeds were successfully removed in all experiments. When the irradiation time was 0.2 s, one of the three experiments was successful. When the irradiation time was 0.1 s, all three experiments were unsuccessful. Therefore, it can be inferred that the minimum time for the 100 W blue laser to remove weeds was between 0.2 s and 0.25 s. When the power was 50 W, the laser irradiated the weeds for 1 s, 0.5 s and 0.4 s, the weeds were successfully removed in all experiments. When the irradiation time was 0.3 s, two of the three experiments were successful. When the irradiation time was 0.25 s, all three experiments failed. Therefore, it can be inferred that the minimum weed removing time of the 50 W blue laser was between 0.3 s and 0.4 s. **Figure 14A** shows some experimental results of removing weeds after irradiating weeds with a 100 W blue laser for 1 s, 0.5 s, and 0.25 s, respectively. **Figure 14B** shows some experimental results of removing weeds after irradiating weeds with a 50 W blue laser for 1 s, 0.5 s, and 0.4 s, respectively.

**Table 8:** Experimental results of weed control with blue lasers of different powers.

Power (W)	Irradiation time (s)	Test 1	Test 2	Test 3	Success rate (%)
100	1	✓	✓	✓	100
	0.5	✓	✓	✓	100
	0.25	✓	✓	✓	100
	0.2	✓	✕	✕	33.3
	0.1	✕	✕	✕	0
50	1	✓	✓	✓	100
	0.5	✓	✓	✓	100
	0.4	✓	✓	✓	100
	0.3	✓	✓	✕	33.3
	0.25	✕	✕	✕	0

✓ indicates that the experiment is successful, and × indicates that the experiment is failed.

**Table 9** describes the removing ability of the 50 W blue lasers for large weeds. By gradually decreasing the irradiation time, it was found that when the time was 0.5 s, two experiment was not able to remove the weeds. Therefore, it can be concluded that the minimum time for the 50 W blue laser to remove large weeds was between 0.5 s and 0.6 s.

**Table 9:** 50 W blue laser removing capacity of large weeds with different irradiation times.

Power (W)	Irradiation time (s)	Test
50	1	✓
	1	✓
	0.8	✓
	0.7	✓
	0.6	✓
	0.5	✕
	0.5	✕

✓ indicates that the experiment is successful, and × indicates that the experiment is failed.

Comparison of NIR and blue lasers in weed control experiments revealed that the weed control effect of a 50 W blue laser was basically the same as that of a 100 W NIR laser. Therefore, as far as NIR and blue lasers were concerned, blue lasers would be more suitable for laser weed control.

### 3.4 Prototype

**Figure 15:**
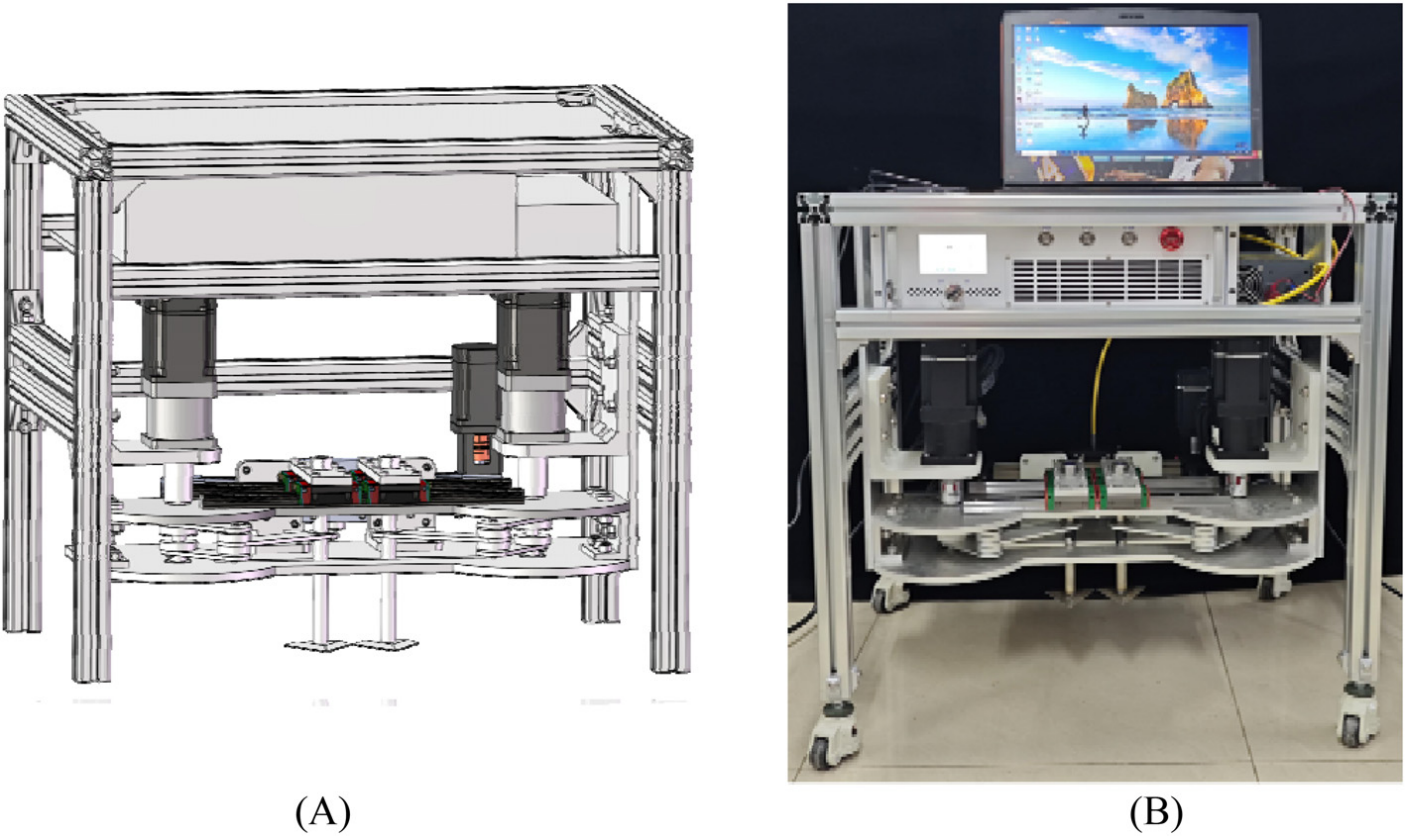
Prototype of mechanical-laser cooperative intra-row weeding device. **(A)** 3D Model, **(B)** First generation prototype.

**Figure 15** shows the first-generation prototype of the mechanical-laser collaborative intra-row weeding device. Technologies such as machining, welding, laser cutting, and 3D printing were used in the processing of the device. In addition, some standard parts, such as guide rail slider modules, aluminum profile, screws, nuts and synchronous belt modules, were also purchased. Among all the machined parts, the weeding knife was made of 304 stainless steels, and the others were made of aluminum alloy. The weeding device was fixed to a body constructed from 40-type aluminum profiles. The laser used fiber output mode. The output end of the laser (laser head) was a focusing lens with an irradiation distance of 120-130 mm and a spot size of 1 mm. The power of the laser was 50 W. The edge angle of the weeding knife was 30° and the soil penetration angle was 0°. The crank length in the prototype was 87 mm, and the link length was 135 mm.

## 4 Discussion

### 4.1 Algorithms and simulations

The optimization of the slider-crank mechanism is an important part of structural design. Many scholars will propose targeted optimization methods (algorithms) for slider cranks based on actual needs. In this research, starting from the basic formula of the slider-crank mechanism, the relationship between the crank, link, working space, and transmission angle was derived. A size optimization algorithm for the slider-crank mechanism was proposed based on these relationships. The algorithm was developed in MATLAB software. The results obtained by the algorithm are often not integers. Many results meet the design requirements but do not meet the processing requirements. To this end, the algorithm was further optimized. When the algorithm obtains the optimization result for the first time, the result will not be output. Instead, the result will be rounded off to verify whether it meets the design requirements, and the optimization result that meets the design requirements will be output. In this way, the crank and link lengths calculated by the optimization algorithm meet the design and processing requirements. In the study, ADAMS was used to perform kinematic simulation verification on the crank slider mechanism. The simulation results were basically consistent with the algorithm operation results. The slight deviation may be due to the retention of two valid values when setting the simulation parameters. This algorithm considers the crank, link, transmission angle, and working space of the slider-crank mechanism. These four factors mainly affect the kinematic characteristics of the slider-crank mechanism, while the factors affecting the dynamic characteristics of the slider-crank mechanism, such as vibration force, vibration torque, and clearance, are not considered in this study (Chaudhary and Chaudhary, 2015; Chen et al., 2021; Huang et al., 2010). Compared with the fields of engines, the application of the slider-crank mechanism in the weeding field is not high-frequency and delicate. Therefore, under the condition of limited resources, only considering the kinematic characteristics will not have a great impact on the weeding robot. When iterating the structural design of the weeding robot, you can consider conducting deeper research.

Analyzing the interaction between soil and farm tools is helpful to improve farming efficiency. The use of simulation software to simulate and analyze the interaction between farm tools and soil is an effective way to reduce experimental costs. This study uses EDEM software to simulate and analyze the force conditions of triangular weeding knives with different soil penetration angles and edge angles in the soil. According to the simulation results, the soil penetration angle of the weeding knife of the mechanical-laser collaborative intra-row weeding robot was determined to be 0° and the edge angle was 30°. The accuracy of simulation results is closely related to the setting of soil parameters. In most of the current related studies, soil parameters are experimentally measured (Yang et al., 2024; Zhang P. et al., 2023, Zhang S. et al., 2023). However, the weeding device will perform weeding in different vegetable fields, and the soil parameters of different vegetable fields are different. For weeding knives, the soil parameters measured experimentally cannot fully represent the soil parameters in actual work. Therefore, this study did not use the experimental measurement method to obtain the soil parameters in the EDEM software, but instead used the literature review method to summarize the soil parameters in the literature published in recent years to determine the soil parameters of this study. When the entire weeding robot is developed, we will use force sensors to conduct field tests to test the force conditions of weeding knives with different soil penetration angles and edge angles.

### 4.2 Laser weed control experiment

In this study, NIR and blue lasers were used to conduct laser weed control experiments in a laboratory environment, and the effects of wavelength, irradiation time and power on the laser weed control effect were explored. Wang, M. et al. (2022) used a 90 W NIR laser to irradiate weeds for 0.64 s to effectively control weeds. In this experiment, a 100 W NIR laser takes about 0.4 s to stably and effectively control weeds. Considering the differences between laboratory and field environments, the results of the laser weeding experiment in this study have a great reference basis. The success of conventional laser weeding experiments is judged based on the change in dry weight of weeds after laser irradiation (Zhu et al., 2022). The success of the weeding experiment in this study was judged when all the leaves of the weeds fell off or the stems of the weeds were burned. The laser weeding method used in this study is consistent with the laser weeding robot of CARBON ROBOTICS, both of which require causing significant damage to the weeds or even killing them. Therefore, although field laser weed control experiments have not been conducted, the possibility of effectively controlling weeds in the field using the laser parameters determined by this experiment is very high. Even if the weed control effect in the laboratory cannot be achieved, it will have a serious impact on the growth ability of weeds. Once the weeding robot is developed, laser weeding experiments will be conducted in vegetable fields to further optimize the laser parameters.

### 4.3 Energy, cost and environment

The laser dose was determined by the irradiation time (*s*) and power (*W*), and the energy consumption was calculated using Equation 8 (Andreasen et al., 2024a, 2024b). The experimental results of this study show that when the NIR laser has a power of 100 W, it requires an energy of 9.55 ${\text{J mm}}^{-2}$ - 12.73 ${\text{J mm}}^{-2}$ to stably and effectively control weeds, while when the blue laser has a power of 50 W, it requires an energy of 4.77 ${\text{J mm}}^{-2}$ - 6.36 ${\text{J mm}}^{-2}$ to stably and effectively control weeds. The irradiation time of both lasers was 0.3-0.4 s. This shows that *Portulaca oleracea L* has a better absorption effect on the wavelength of 450 nm than the combined wavelength of 915 and 918 nm. The efficiency of laser weed control is also related to the ability of weeds to absorb wavelength. Under the same circumstances, blue lasers are more energy efficient.


(8) $$\text{Dose}\left({\text{J mm}}^{-2}\right)=W\times s/\left(22/7\times 1^{2}mm^{2}\right)$$

The current mainstream laser weeding robots use CO_2_ lasers, NIR lasers and blue lasers. This study consulted five laser production companies in China (Beijing, Shenzhen, Shanghai and Changchun) and a British laser company. According to consultation, the price of a 100 W NIR (wavelength: approximately 980 nm) laser is about 20,000-35,000 RMB, the price of a 50 W blue laser (wavelength: approximately 450 nm) is 25,000-35,000 RMB, the price of a 150 W CO_2_ glass tube laser (wavelength: 10.6 μm) is about 3,000 RMB and the price of a 150 W CO_2_ radio frequency laser (wavelength: 10.6 μm) is about 40,000 RMB. In terms of size, the length, width and height of the blue and NIR lasers are approximately 50 cm × 50 cm × 15 cm (air cooling mode), width and height of the CO_2_ radio frequency laser approximately 66 cm × 20 cm × 22 cm, and the CO_2_ glass tube laser is a cylinder about 170 cm long and 8 cm in diameter. The CO_2_ glass tube laser is the cheapest of the four lasers, but also the longest. The other four lasers are packaged by laser manufacturers, so they are relatively expensive. Considering both weed control efficiency and robot cost, buying the accessories and making them yourself is a feasible idea. In addition, selecting a wavelength that is more efficiently absorbed by weeds, such as 450 nm in this study (compared to 980 and 915 nm), can reduce energy consumption under the same conditions. In the future, as laser technology develops, the performance of lasers will get better and better while the price will also decrease.

Both laser weeding and mechanical weeding are environmentally friendly (Krupanek et al., 2024). If the laser weeding robot wants to be able to handle high-density fields, it needs to use multiple laser modules. For example, the laser weeding robot developed by CARBON ROBOTICS uses 32 lasers. But this means high cost and high energy consumption. Compared with laser weeding robots, mechanical weeding robots have lower manufacturing costs and consume less energy. However, they are more likely to damage crops when removing weeds very close to them. Therefore, most mechanical weeding robots currently set up a safety circle around the crops. The mechanical-laser collaborative intra-row weeding prototype designed in this study is a new weeding device that combines the advantages of mechanical weeding and laser weeding. It can further improve the removal rate of intra-row weeds while ensuring the integrity of the crop. With the development of renewable energy such as solar energy, the energy cost of weeding robots will no longer be a problem.

### 4.4 Mechanical-laser collaborative intra-row weeding device

At present, bionic agricultural tools are a hot research direction (Luo et al., 2024). Compared with the traditional ridger, the bionic ridger designed based on the shape of a wild boar head reduced the penetration resistance by 16.67% (laboratory test) at a speed of 4.2 km/h, and the penetration resistance decreased by 6.91% in field tests (Li et al., 2017; Salem et al., 2021). The bionic electro-osmosis technology inspired by the body surface of burrowing animals can reduce the soil adhesion of tillage tools by 29.8%-90% (Massah et al., 2021). Salem et al. (2021) designed and tested 27 domed discs inspired by soil burrowing animals to determine the optimal dimensions of the dome surface to reduce drag. Tests were conducted under laboratory conditions and the results showed that a properly designed dome surface can significantly reduce drag in cohesive soils compared to a flat disc. Many studies have shown that agricultural tools designed based on bionics have better performance than traditional agricultural tools. However, agricultural tools based on bionic design often have higher processing costs than traditional agricultural tools. This study used a traditional triangular weeding knife, which has the advantage of low processing cost. In the future, when upgrading and iterating the structural design of the weeding robot, finding some animals with bionic shapes like triangles will be consider developing bionic weeding knives. Perhaps this is a way to balance the performance and manufacturing cost of the weeding knife.

Through the design and optimization of the structure, this study realized the integrated structural design of mechanical weeding and laser weeding and manufactured the first prototype. To ensure the structural strength of the entire device, stainless steel and aluminum alloy were mainly used in processing and manufacturing. In addition, our developed a lightweight deep learning algorithm with intra-row weed severity classification capabilities (Hu et al., 2024). The algorithm classifies the severity of the intra-row weeds three levels (no weeds, mild and severe), solving the problem of how mechanical weeding and laser weeding can work together. Currently, the control system is under development. After the control system development and weed control device debugging are completed, weed control tests will be carried out in the lettuce field. This field test intends to test the actual weeding performance of the weeding device through three modes: simple mechanical weeding mode, laser weeding mode, and mechanical-laser collaborative weeding mode. Based on the test results, the weeding device will be upgraded by lightweight design, improved control system, optimized identification and positioning system, and bionic weeding knife design.

## 5 Conclusions

In this study, a novel mechanical-laser collaborative weeding device structure was proposed for weed control in vegetable intra-row. A slider-crank mechanism size optimization algorithm was proposed for the design and optimization of the slider-crank mechanism. When the initial parameters *γ*_min1_, *γ*_min2_, *γ*_min_, *γ*_max1_, *γ*_max2_, *S* were 49°, 51°, 50°, 60°, 70° and 120 mm, respectively. *L*_1_, *L*_2_, *θ*_truth_, *γ*_mint_ and *γ*_maxt_ were 87 mm, 135 mm, 46.397187°, 49.875965° and 60.717813°, respectively. To verify the correctness of the algorithm, the kinematics of the crank slider mechanism was simulated using ADAMS software, and the results were *γ*_mint_ = 50.067°, *γ*_maxt_ = 60.1558°, and *S* = 120.0085 mm. The simulation results were basically consistent with the algorithm results. EDEM software was used to simulate and analyze the resistance of triangular weeding knives with different soil penetration angles and edge angles in the soil. The simulation results show that the weeding knife encountered the least resistance when its soil penetration angle was 0° and its edge angle was 30°. In addition, weed removal experiments with different powers and lasers were conducted using a 200 W NIR and 200 W blue laser. The experimental results show that the time it took for a 50 W blue laser and a 100 W NIR laser to remove small weeds was approximately between 0.3 and 0.4 s, and the time it took for a 50 W blue laser to remove larger weeds was approximately between 0.5 and 0.6 s. The time it took for 75 W and 50 W NIR lasers to remove weeds was greater than 1 s. A 50 W blue laser is a good laser for real-time laser weed control. Based on the above research results, a prototype of a mechanical-laser collaborative intra-row weeding device was built. The main processing technologies used were machining, laser cutting, and 3D printing. The weeding device used a 50 W blue laser with a spot size of 1 mm and a focusing lens irradiation distance of 120-130 mm. The edge angle of the weeding knife used was 30°, and the soil penetration angle was 0°. The crank and link lengths were 87 mm and 135 mm, respectively. The mechanical-laser collaborative intra-row weeding method proposed in this study provides a new idea in the field of intelligent weeding. The relevant experimental results can provide a reference for the design of mechanical weeding equipment and laser weeding equipment design.

## Data Availability

The original contributions presented in the study are included in the article/supplementary materials. Further inquiries can be directed to the corresponding author.

## References

[B1] Aikins K. A. Antille D. L. Ucgul M. Barr J. B. Jensen T. A. Desbiolles J. M.A. (2021a). Analysis of effects of operating speed and depth on bentleg opener performance in cohesive soil using the discrete element method. Comput. Electron. Agric. 187, 106236. doi: 10.1016/j.compag.2021.106236

[B2] Aikins K. A. Barr J. B. Antille D. L. Ucgul M. Jensen T. A. Desbiolles J. M.A. (2021b). Analysis of effect of bentleg opener geometry on performance in cohesive soil using the discrete element method. Biosyst. Eng. 209, 106–124. doi: 10.1016/j.biosystemseng.2021.06.007

[B3] Aikins K. A. Ucgul M. Barr J. B. Jensen T. A. Antille D. L. Desbiolles J. M.A. (2021c). Determination of discrete element model parameters for a cohesive soil and validation through narrow point opener performance analysis. Soil Tillage Res. 213, 105123. doi: 10.1016/j.still.2021.105123

[B4] Alanaz A. R. Alatawi E. A.S. Alotaibi R. S. Alatawi E. A.H. Albalawi A. D. Alhumayri H. A.. (2023). The Bio-herbicidal potential of some wild plants with allelopathic effects from Tabuk Region on selected local weed species. Front. Plant Sci. 14. doi: 10.3389/fpls.2023.1286105 PMC10739508 38143576

[B5] Andreasen C. Scholle K. Saberi M. (2022). Laser weeding with small autonomous vehicles: friends or foes? Front. Agron. 4. doi: 10.3389/fagro.2022.841086

[B6] Andreasen C. Vlassi E. Salehan N. (2024a). Laser weeding of common weed species. Front. Plant Sci. 15. doi: 10.3389/fpls.2024.1375164 PMC11157096 38855471

[B7] Andreasen C. Vlassi E. Salehan N. (2024b). Laser weeding: opportunities and challenges for couch grass (Elymus repens (L.) Gould) control. Sci. Rep. 14, 11173. doi: 10.1038/s41598-024-61742-8 38750179 PMC11096317

[B8] Awuah E. Zhou J. Liang Z. Aikins K. A. Gbenontin B. V. Mecha P.. (2022). Parametric analysis and numerical optimisation of Jerusalem artichoke vibrating digging shovel using discrete element method. Soil Tillage Res. 219, 105344. doi: 10.1016/j.still.2022.105344

[B9] Bawden O. Kulk J. Russell R. McCool C. English A. Dayoub F.. (2017). Robot for weed species plant-specific management. J. Field Robotics 34, 1179–11995. doi: 10.1002/rob.21727

[B10] Chaudhary K. Chaudhary H. (2015). Optimal dynamic balancing and shape synthesis of links in planar mechanisms. Mech. Mach. Theory 93, 127–146. doi: 10.1016/j.mechmachtheory.2015.07.006

[B11] Chen Y. Wu K. Wu X. Sun Y. Zhong T. (2021). Kinematic accuracy and nonlinear dynamics of a flexible slider-crank mechanism with multiple clearance joints. Eur. J. Mechanics - A/Solids 88, 104277. doi: 10.1016/j.euromechsol.2021.104277

[B12] Dedousis A. P. Godwin R. J. (2008). “The rotating disc-hoe – an overview of the system for mechanical weed control,” in 2008 Providence, Rhode Island, St. Joseph, MI, June 29 – July 2, 2008 (St. Joseph, Michigan: American Society of Agricultural and Biological Engineers). Available at: https://elibrary.asabe.org/abstract.asp?aid=24585&t=5.

[B13] El-Mastouri Z. Košnarová P. Hamouzová K. Alimi E. Soukup J. (2024). Insight into the herbicide resistance patterns in Lolium rigidum populations in Tunisian and Moroccan wheat regions. Front. Plant Sci. 15. doi: 10.3389/fpls.2024.1331725 PMC10877012 38379946

[B14] Fang H. Niu M. Wang X. Zhang Q. (2022). Effects of reduced chemical application by mechanical-chemical synergistic weeding on maize growth and yield in East China. Front. Plant Sci. 13. doi: 10.3389/fpls.2022.1024249 PMC9549763 36226290

[B15] Hu R. Su W.-H. Li J.-L. Peng Y. (2024). Real-time lettuce-weed localization and weed severity classification based on lightweight YOLO convolutional neural networks for intelligent intra-row weed control. Comput. Electron. Agric. 226, 109404. doi: 10.1016/j.compag.2024.109404

[B16] Huang M.-S. Chen K.-Y. Fung R.-F. (2010). Comparison between mathematical modeling and experimental identification of a spatial slider–crank mechanism. Appl. Math. Model. 34, 2059–20735. doi: 10.1016/j.apm.2009.10.018

[B17] Jiang B. Zhang J.-L. Su W.-H. Hu R. (2023). A SPH-YOLOv5x-based automatic system for intra-row weed control in lettuce. Agronomy 13, 2915. doi: 10.3390/agronomy13122915

[B18] Jiao J. Hu L. Chen G. Chen C. Zang Y. (2024). Development and experimentation of intra-row weeding device for organic rice. Agriculture 14, 146. doi: 10.3390/agriculture14010146

[B19] Ju J. Chen G. Lv Z. Zhao M. Sun L. Wang Z.. (2024). Design and experiment of an adaptive cruise weeding robot for paddy fields based on improved YOLOv5. Comput. Electron. Agric. 219, 108824. doi: 10.1016/j.compag.2024.108824

[B20] Kim Y.-S. Siddique M. A. A. Kim W.-S. Kim Y.-J. Lee S.-D. Lee D.-K.. (2021). DEM simulation for draft force prediction of moldboard plow according to the tillage depth in cohesive soil. Comput. Electron. Agric. 189, 106368. doi: 10.1016/j.compag.2021.106368

[B21] Krupanek J. de Santos P. G. Emmi L. Wollweber M. Sandmann H. Scholle K.. (2024). Environmental performance of an autonomous laser weeding robot—a case study. Int. J. Life Cycle Assess. 29, 1021–10525. doi: 10.1007/s11367-024-02295-w

[B22] Langsenkamp F. Sellmann F. Kohlbrecher M. Kielhorn A. Strothmann W. Michaels A.. (2014). "Tube Stamp for mechanical intra-row individual Plant Weed Control." in Proceedings of the 18th World Congress of CIGR (Beijing, China) 2014, 16–19.

[B23] Li J. Yan Y. Chirende B. Wu X. Wang Z. Zou M. (2017). Bionic design for reducing adhesive resistance of the ridger inspired by a boar’s head. Appl. Bionics Biomech. 2017, 8315972. doi: 10.1155/2017/8315972 28757796 PMC5512032

[B24] Li J.-L. Su W.-H. Zhang H.-Y. Peng Y. (2023). A real-time smart sensing system for automatic localization and recognition of vegetable plants for weed control. Front. Plant Sci. 14. doi: 10.3389/fpls.2023.1133969 PMC10083263 37051077

[B25] Liu L. Wang X. Zhang X. Zhong X. Wei Z. Geng Y.. (2023). Determination and verification of parameters for the discrete element modelling of single disc covering of flexible straw with soil. Biosyst. Eng. 233, 151–167. doi: 10.1016/j.biosystemseng.2023.08.001

[B26] Liu L. Wu L. Li Z. Fang Y. Ju B. Zhang S.. (2024). The Pro-197-Thr mutation in the ALS gene confers novel resistance patterns to ALS-inhibiting herbicides in Bromus japonicus in China. Front. Plant Sci. 15. doi: 10.3389/fpls.2024.1348815 PMC10917945 38455726

[B27] Luo Y. Li J. Yao B. Luo Q. Zhu Z. Wu W. (2024). Research progress and development trend of bionic harvesting technology. Comput. Electron. Agric. 222, 109013. doi: 10.1016/j.compag.2024.109013

[B28] Makange N. R. Ji C. Torotwa I. (2020). Prediction of cutting forces and soil behavior with discrete element simulation. Comput. Electron. Agric. 179, 105848. doi: 10.1016/j.compag.2020.105848

[B29] Marx C. Barcikowski S. Hustedt M. Haferkamp H. Rath T. (2012). Design and application of a weed damage model for laser-based weed control. Biosyst. Eng. 113, 148–1575. doi: 10.1016/j.biosystemseng.2012.07.002

[B30] Massah J. Fard M. R. Aghel H. (2021). An optimized bionic electro-osmotic soil-engaging implement for soil adhesion reduction. J. Terramechanics 95, 1–6. doi: 10.1016/j.jterra.2021.01.003

[B31] Michaliszyn-Gabryś B. Bronder J. Jarosz W. Krupanek J. (2024a). Potential of eco-weeding with high-power laser adoption from the farmers’ perspective. Sustainability 16, 2353. doi: 10.3390/su16062353

[B32] Michaliszyn-Gabryś B. Bronder J. Krupanek J. (2024b). Social life cycle assessment of laser weed control system: A case study. Sustainability 16, 2590. doi: 10.3390/su16062590

[B33] Parasca S. C. Spaeth M. Rusu T. Bogdan I. (2024). Mechanical weed control: sensor-based inter-row hoeing in sugar beet (Beta vulgaris L.) in the transylvanian depression. Agronomy 14, 176. doi: 10.3390/agronomy14010176

[B34] Pérez-Ruíz M. Slaughter D. C. Fathallah F. A. Gliever C. J. Miller B. J. (2014). Co-robotic intra-row weed control system. Biosyst. Eng. 126, 45–55. doi: 10.1016/j.biosystemseng.2014.07.009

[B35] Pingarron-Cardenas G. Onkokesung N. Goldberg-Cavalleri A. Lange G. Dittgen J. Edwards R. (2024). Selective herbicide safening in dicot plants: a case study in Arabidopsis. Front. Plant Sci. 14. doi: 10.3389/fpls.2023.1335764 PMC10822893 38288413

[B36] Qin L. Xu Z. Wang W. Wu X. (2024). YOLOv7-based intelligent weed detection and laser weeding system research: targeting veronica didyma in winter rapeseed fields. Agriculture 14, 910. doi: 10.3390/agriculture14060910

[B37] Quan L. Jiang W. Li H. Li H. Wang Q. Chen L. (2022). Intelligent intra-row robotic weeding system combining deep learning technology with a targeted weeding mode. Biosyst. Eng. 216, 13–31. doi: 10.1016/j.biosystemseng.2022.01.019

[B38] Raja R. Nguyen T. T. Slaughter D. C. Fennimore S. A. (2020a). Real-time weed-crop classification and localisation technique for robotic weed control in lettuce. Biosyst. Eng. 192, 257–274. doi: 10.1016/j.biosystemseng.2020.02.002

[B39] Raja R. Nguyen T. T. Vuong V. L. Slaughter D. C. Fennimore S. A. (2020b). RTD-SEPs: Real-time detection of stem emerging points and classification of crop-weed for robotic weed control in producing tomato. Biosyst. Eng. 195, 152–171. doi: 10.1016/j.biosystemseng.2020.05.004

[B40] Rakhmatulin I. Andreasen C. (2020). A concept of a compact and inexpensive device for controlling weeds with laser beams. Agronomy 10, 1616. doi: 10.3390/agronomy10101616

[B41] Saber M. Lee W. S. Burks T. F. Schueller J. K. Chase C. A. MacDonald G. E.. (2015). “Performance and evaluation of intra-row weeder ultrasonic plant detection system and pinch-roller weeding mechanism for vegetable crops,” in 2015 ASABE Annual International Meeting (St. Joseph, MI: American Society of Agricultural and Biological Engineers). Available at: https://elibrary.asabe.org/abstract.asp?aid=46319&t=5. doi: 10.13031/aim.20152188868

[B42] Sahin Y. Z. Cay A. (2024). Depressive effects of diode laser on selected weeds in field conditions. Appl. Ecol. Environ. Res. 22, 3677–3690. doi: 10.15666/aeer/2204_36773690

[B43] Salem A. E. Wang H. Gao Y. Zha X. Abdeen M. A. Zhang G. (2021). Effect of biomimetic surface geometry, soil texture, and soil moisture content on the drag force of soil-touching parts. Appl. Sci. 11, 8927. doi: 10.3390/app11198927

[B44] Saunders C. Ucgul M. Godwin R. J. (2021). Discrete element method (DEM) simulation to improve performance of a mouldboard skimmer. Soil Tillage Res. 205, 104764. doi: 10.1016/j.still.2020.104764

[B45] Sellmann F. Bangert W. Grzonka S. Hänsel M. Haug S. Kielhorn A.. (2014). "RemoteFarming.1: Human-machine interaction for a field-robot-based weed control application in organic farming" in 4th International Conference on Machine Control & Guidance 2014, 19–20.

[B46] Shi Y. Xin S. Wang X. Hu Z. Newman D. Ding W. (2019). Numerical simulation and field tests of minimum-tillage planter with straw smashing and strip laying based on EDEM software. Comput. Electron. Agric. 166, 105021. doi: 10.1016/j.compag.2019.105021

[B47] Song W. Jiang X. Li L. Ren L. Tong J. (2022). Increasing the width of disturbance of plough pan with bionic inspired subsoilers. Soil Tillage Res. 220, 105356. doi: 10.1016/j.still.2022.105356

[B48] Sun J. Chen H. Wang Z. Ou Z. Yang Z. Liu Z.. (2020). Study on plowing performance of EDEM low-resistance animal bionic device based on red soil. Soil Tillage Res. 196, 104336. doi: 10.1016/j.still.2019.104336

[B49] Tillett N. D. Hague T. Grundy A. C. Dedousis A. P. (2008). Mechanical within-row weed control for transplanted crops using computer vision. Biosyst. Eng. 99, 171–178. doi: 10.1016/j.biosystemseng.2007.09.026

[B50] Tudi M. Daniel Ruan H. Wang L. Lyu J. Sadler R. Connell D.. (2021). Agriculture development, pesticide application and its impact on the environment. Int. J. Environ. Res. Public Health 18, 1112. doi: 10.3390/ijerph18031112 33513796 PMC7908628

[B51] Wang M. Leal-Naranjo J.-A. Ceccarelli M. Blackmore S. (2022). A novel two-degree-of-freedom gimbal for dynamic laser weeding: design, analysis, and experimentation. IEEE/ASME Trans. Mechatronics 27, 5016–50265. doi: 10.1109/tmech.2022.3169593

[B52] Wang Y. Xue W. Ma Y. Tong J. Liu X. Sun J. (2019). DEM and soil bin study on a biomimetic disc furrow opener. Comput. Electron. Agric. 156, 209–216. doi: 10.1016/j.compag.2018.11.023

[B53] Wang X. Zhang Q. Huang Y. Ji J. (2022). An efficient method for determining DEM parameters of a loose cohesive soil modelled using hysteretic spring and linear cohesion contact models. Biosyst. Eng. 215, 283–294. doi: 10.1016/j.biosystemseng.2022.01.015

[B54] Wang X. Zhang S. Pan H. Zheng Z. Huang Y. Zhu R. (2019). Effect of soil particle size on soil-subsoiler interactions using the discrete element method simulations. Biosyst. Eng. 182, 138–150. doi: 10.1016/j.biosystemseng.2019.04.005

[B55] Wang Y. Zhang D. Yang L. Cui T. Jing H. Zhong X. (2020). Modeling the interaction of soil and a vibrating subsoiler using the discrete element method. Comput. Electron. Agric. 174, 105518. doi: 10.1016/j.compag.2020.105518

[B56] Wu X. Aravecchia S. Lottes P. Stachniss C. Pradalier C. (2020). Robotic weed control using automated weed and crop classification. J. Field Robotics 37, 322–3405. doi: 10.1002/rob.21938

[B57] Xie Q. Song M. Wen T. Cao W. Zhu Y. Ni J. (2024). An intelligent spraying system for weeds in wheat fields based on a dynamic model of droplets impacting wheat leaves. Front. Plant Sci. 15. doi: 10.3389/fpls.2024.1420649 PMC11211531 38947943

[B58] Yang L. Li J. Lai Q. Zhao L. Li J. Zeng R.. (2024). Discrete element contact model and parameter calibration for clayey soil particles in the Southwest hill and mountain region. J. Terramechanics 111, 73–87. doi: 10.1016/j.jterra.2023.10.002

[B59] Yu Z. He X. Qi P. Wang Z. Liu L. Han L.. (2024). A static laser weeding device and system based on fiber laser: development, experimentation, and evaluation. Agronomy 14, 1426. doi: 10.3390/agronomy14071426

[B60] Zhang H. Cao D. Zhou W. Currie K. (2024). Laser and optical radiation weed control: a critical review. Precis. Agric. 25, 2033–20575. doi: 10.1007/s11119-024-10152-x

[B61] Zhang P. Li F. Wang F. (2023). Optimization and test of ginger-shaking and harvesting device based on EDEM software. Comput. Electron. Agric. 213, 108257. doi: 10.1016/j.compag.2023.108257

[B62] Zhang Z. Wang H. Gu T. Cao J. Lou Y. Li G. (2023). Propyrisulfuron plus cyhalofop butyl as one-shot herbicides provide high weed control efficiency and net economic performance in mechanically transplanted rice. Front. Plant Sci. 14. doi: 10.3389/fpls.2023.1281931 PMC10619164 37920722

[B63] Zhang L. Zhai Y. Chen J. Zhang Z. Huang S. (2022). Optimization design and performance study of a subsoiler underlying the tea garden subsoiling mechanism based on bionics and EDEM. Soil Tillage Res. 220, 105375. doi: 10.1016/j.still.2022.105375

[B64] Zhang L. Zhai Y. Wu C. Huang S. Zhang Z. (2023). Modeling the interaction between a new four-bar subsoiling mechanism and red soil using the improved differential evolution algorithm and DEM. Comput. Electron. Agric. 208, 107783. doi: 10.1016/j.compag.2023.107783

[B65] Zhang S. Zhao H. Wang X. Dong J. Zhao P. Yang F.. (2023). Discrete element modeling and shear properties of the maize stubble-soil complex. Comput. Electron. Agric. 204, 107519. doi: 10.1016/j.compag.2022.107519

[B66] Zhao J. Lu Y. Wang X. Zhuang J. Han Z. (2023). A bionic profiling-energy storage device based on MBD-DEM coupled simulation optimization reducing the energy consumption of deep loosening. Soil Tillage Res. 234, 105824. doi: 10.1016/j.still.2023.105824

[B67] Zhu H. Zhang Y. Mu D. Bai L. Zhuang H. Li H. (2022). YOLOX-based blue laser weeding robot in corn field. Front. Plant Sci. 13. doi: 10.3389/fpls.2022.1017803 PMC9674089 36407588

